# Performance Enhancing Diets and the PRISE Protocol to Optimize Athletic Performance

**DOI:** 10.1155/2015/715859

**Published:** 2015-04-20

**Authors:** Paul J. Arciero, Vincent J. Miller, Emery Ward

**Affiliations:** ^1^Human Nutrition and Metabolism Laboratory, Health and Exercise Sciences Department, Skidmore College, Saratoga Springs, NY 12866, USA; ^2^College of Graduate Health Studies, A. T. Still University, Mesa, AZ 85206, USA

## Abstract

The training regimens of modern-day athletes have evolved from the sole emphasis on a single fitness component (e.g., endurance athlete or resistance/strength athlete) to an integrative, multimode approach encompassing all four of the major fitness components: resistance (R), interval sprints (I), stretching (S), and endurance (E) training. Athletes rarely, if ever, focus their training on only one mode of exercise but instead routinely engage in a multimode training program. In addition, timed-daily protein (P) intake has become a hallmark for all athletes. Recent studies, including from our laboratory, have validated the effectiveness of this multimode paradigm (RISE) and protein-feeding regimen, which we have collectively termed PRISE. Unfortunately, sports nutrition recommendations and guidelines have lagged behind the PRISE integrative nutrition and training model and therefore limit an athletes' ability to succeed. Thus, it is the purpose of this review to provide a clearly defined roadmap linking specific performance enhancing diets (PEDs) with each PRISE component to facilitate optimal nourishment and ultimately optimal athletic performance.

## 1. Introduction

At every level of athletic competition, the drive to succeed is a natural competitive instinct that requires an appropriate amount, type, and timing of exercise training and nutrient intake. This balance is important because the difference between winning and losing largely depends on the training and nutritional status of the athlete. Thus, in order for any athlete to be successful, proper training and nourishment must be a daily priority.

Specific training regimens for elite athletes are often based on the same science used to formulate exercise and nutrition recommendations for the general public. For example, governing organizations in sports medicine (American College of Sports Medicine, ACSM) and healthcare (American Heart Association, AHA; Centers for Disease Control, CDC; World Health Organization, WHO) generally promote an exercise regimen that includes a combination of (i) cardiorespiratory (aerobic) (150 minutes/week of 30–60 minutes moderate-intensity 5 days/week or 20–60 minutes vigorous-intensity exercise 3 days/week); (ii) resistance (major muscle groups 2-3 days/week of 2–4 sets and 8–20 repetitions); (iii) flexibility (stretches held for 10–30 seconds, repeated 2–4 times 2-3 days/week); and (iv) neuromotor/functional exercise (balance, agility, coordination 20–30 minutes/day 2-3 days/week).

While the intent of these exercise recommendations is noble, the majority of the US population (>60%) falls short in achieving them [1–3], especially among youth. It may very well be the case, exercise compliance and adherence suffers because the current recommendations are not realistic (up to 7 days of exercise per week) or compatible with many lifestyles. An additional concern with current exercise guidelines is they often lack a clear and specific connection to appropriate dietary intake recommendations.

**Figure 1:**
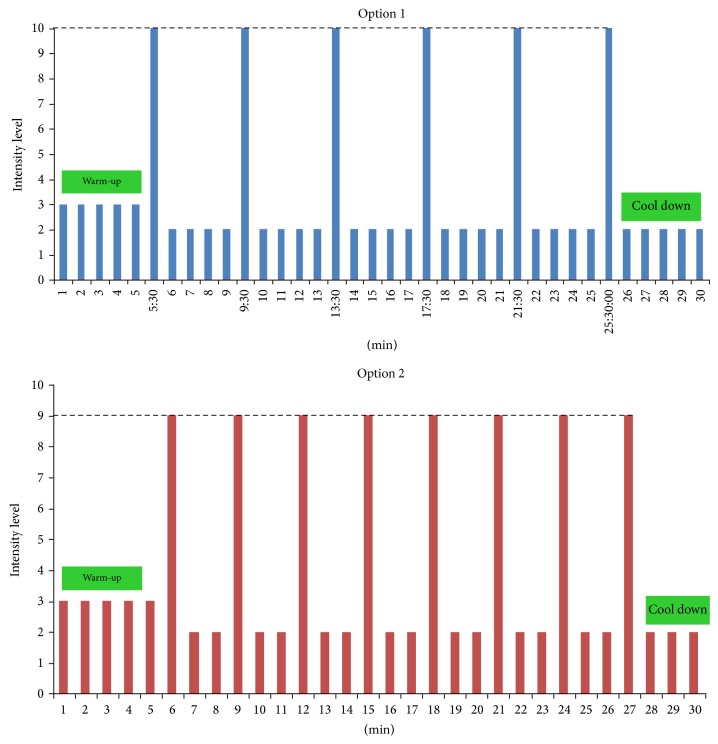
Interval exercise (I). Choose an exercise (walking, jogging, running, cycling, swimming, elliptical, snowshoeing, cross-country skiing, jumping rope, rollerblading, rowing, etc.) and one of two options. Option 1: perform 5–7 “all-out” sprint Intervals for 30-seconds at intensity level 10 followed by a 4 minute recovery at intensity Level 2; or Option 2: perform 8–12 sprint “almost all-out” intervals for 60 seconds at intensity level 9 followed by a 2-minute recovery at intensity Level 2. At the beginning and end of each interval session perform a 5-minute dynamic warm-up and gentle stretching cool down, respectively, so that each session is completed within 30–40 minutes.

**Figure 2:**
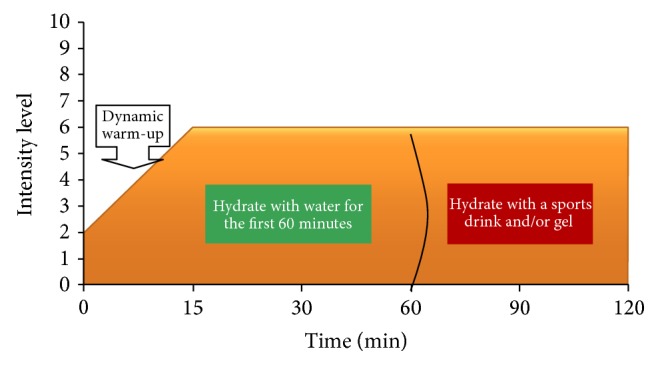
Endurance exercise (E). Perform endurance (E) exercise at an intensity level of 6 for 60 minutes or longer using any form of exercise (walking, jogging, running, cycling, swimming, hiking, cross-country skiing, snowshoeing, rollerblading, rowing, etc.). Ideally, perform E outside in nature and in the morning. At the beginning and end of each E session perform a 5-minute dynamic warm-up and a cool-down gentle stretch, respectively.

Interestingly, the contemporary athlete (competitive and noncompetitive) no longer adheres to the traditional, narrowly defined training regimen focused on only one mode of exercise (e.g., only endurance or only resistance) but instead adheres to a multimode, integrative training model. Indeed, the challenge for most athletes today is finding the balance (time and energy) to incorporate all of the fitness components (resistance, anaerobic, aerobic, and flexibility training) into their regular training regimen, recognizing the vital importance each one contributes to their overall success. Thus, herein we propose a scientifically validated model that embraces a holistic and integrative model of exercise training that all athletes are encouraged to follow, termed “PRISE” (Table 1) [4]. The “P” is timed-daily protein-pacing intake; the “R” is resistance training; “I” is interval anaerobic sprint training; “S” is stretching (flexibility, restorative) training; and “E” is endurance aerobic training and is based on 4 days of structured exercise per week (Tables 2 and 3; Figures 1 and 2). This novel paradigm of exercise training integrates the four major fitness components into the training regimen of all athletes, regardless of sport, while still allowing for an athlete to emphasize sport-specific training.

Perhaps equally, if not more, important for athletic performance is proper nourishment, including the type, timing, and amount of specific food and dietary supplement sources. Currently, there is disconnect between sports nutrition guidelines and the progressive multicomponent exercise training regimen (PRISE) that many athletes follow. As an example, most endurance athletes (marathoners, triathletes, etc.) are encouraged to follow a consistent diet of relatively high carbohydrate intake (60–70% of total kcals). However, most endurance athletes adhere to a PRISE training schedule, including resistance (R), interval (I), and stretching (S) training, and therefore need to adapt their nourishment to match this integrative training paradigm in order to achieve success and the same applies to the sprint-type athlete.

It is clear that our current exercise training and nutrition practices need to be readjusted to meet the needs of the evolving athlete. Thus, the major objective of the current sports nutrition review is to establish a clear rationale and link between a scientifically proven integrative model of exercise training (PRISE) performed four days per week and a matching sports performance enhancing diet (PED), to maximize athletic performance. We advocate following the PRISE protocol and linking the prescribed PED to each component for that day to maximize the physiological, biochemical, and hormonal responses. The advantage of incorporating these nutritional strategies on a temporal basis allows the body to avoid repeated long-term exposure and thus potential for adverse side effects, downregulation (i.e., decreased cellular sensitivity), and tolerance to occur. In addition, athletes should follow a balanced, protein-rich diet that incorporates 20–30 grams of high-quality protein evenly spaced throughout the day (~every 3 hours), including nonexercising days.

## 2. Timed-Daily Protein-Pacing (P) Intake

Protein is arguably the most crucial nutrient for general health and athletic performance because of its role in protein synthesis, energy metabolism, body composition (optimal lean muscle mass and fat mass), immune support, and satiation. Further, research supports timed-daily protein feedings throughout the day to maximize protein synthesis and thus lean muscle mass accretion [5–7]. Dietary guidelines have consistently encouraged a higher carbohydrate (CHO) intake (up to 65% of total kcals), moderate fat (20–35% of total kcals), and 10–35% of intake as protein (PRO) for proper weight control [8]. However, recent data suggests that consuming protein at the higher acceptable range (~25–35%) enhances energy expenditure [9–11] and body composition [4, 7, 12–14] and may do so independent of inducing weight loss [15]. This is important because it will have important implications for athletes attempting to improve health and performance outcomes without undergoing caloric restriction and weight reduction. Recent data also shows that the combined effects of increased dietary PRO and reduced glycemic index (GI) diets enhances weight loss maintenance [16] and improves body composition [17, 18].

Meal frequency (number of meals eaten) is another important factor for optimization of body composition and athletic performance. Several studies have suggested meal frequency is inversely related to body weight [19, 20].

*Mechanisms*. It is well established that energy expenditure and metabolism differ greatly in response to macronutrient intake of isoenergetic meals. For example, protein intake elicits the greatest thermogenic response compared to carbohydrate and fat [21–23] and this may be related to increased satiation [21]. In addition, compelling evidence favors dietary proteins containing a full complement of essential amino acids with a high leucine content to maximally stimulate muscle protein synthesis [24–26]. In this case, whey protein is considered the ideal protein source. Thus, the precise mechanism responsible for enhanced energy expenditure following macronutrient intake is partly due to an increase in muscle protein synthesis (MPS) that is triggered by protein ingestion. In addition, there is speculation that a frequent macronutrient intake, especially protein-containing meals, favors an anabolic state resulting in an increase in protein synthesis and accretion [5, 26]. Specifically, increased meal frequency (timed-ingestion every 3 hours) of 20 gram servings of whey protein maximizes MPS as well as signaling proteins and transcriptional activity of muscle cells [5]. Indeed, not only does this have beneficial implications for increased energy expenditure but also for enhanced functional capacity of muscles and an increase in lean body mass, all of which lead to improved body weight control and athletic performance.

*Evidence*. Our laboratory previously demonstrated that higher PRO (25%, 40%) intakes, including whey protein, more favorably affect body composition compared with a traditional diet (PRO < 20%) consumed over 6 meals per day [12, 13]. In both studies, subjects consuming the higher PRO 6 meals/day lost more body weight, fat mass, and abdominal fat mass and maintained lean body mass. In follow-up to these investigations, our laboratory recently compared a higher PRO (~35% of kcals) diet (containing ~50% whey protein), moderate in CHO (~40% of kcals) consumed at either 3 or 6 meals/day versus a lower PRO (~15% of kcals) diet, higher in CHO (~60% of kcals) consumed at 3 meals/day, both of which contained complex, low-GI (GI values of <50) CHO's consumed throughout 28 days of energy balance (weight maintenance), and deficit (weight loss), respectively (56 days total) [7]. Our results demonstrated that following the 28-day period of energy balance (weight maintenance) total and abdominal body fat decreased and lean body mass (LBM) increased in the higher PRO six meals/day (HP6) group versus the 3 meals/day higher PRO and CHO groups. During the 28-day weight loss period, total and abdominal fat continued to decrease and LBM remained elevated only in HP6.

Perhaps most interesting was the finding that postprandial thermogenesis during both weight maintenance and loss was significantly elevated (67–100%) in HP6 compared to the 3 meals per day groups [7]. The increased thermic response in HP6 may partly explain the enhanced total and abdominal fat loss in this group. These findings indicate that macronutrient composition (increased dietary protein), nutrient quality (low glycemic index and unprocessed carbohydrates), and frequency of eating (6x per day) are more important than total energy intake to enhance body composition (reduce abdominal obesity and maintain lean body mass) and enhance postprandial thermogenesis during both weight maintenance and weight loss.

*Practical Use*. Consuming increased amounts of dietary protein (20–30 grams/serving or 25–35% of total kcal intake), mostly from whey protein sources, more often (4–6 meals meals/day) throughout the day (every 3 hours) decreases abdominal fat and increases postprandial thermogenesis and lean body mass compared to traditional protein and meal frequency intakes. These body composition changes may directly lead to enhanced athletic performance. Importantly, these beneficial improvements are achieved even though total kcals consumed are identical to a traditional feeding pattern. The data from our laboratory indicate, for the first time, that macronutrient composition (increased dietary protein), nutrient quality (low glycemic index and unprocessed carbohydrates), and frequency of eating (4–6x per day) are more important than total energy intake to improve body composition and postprandial thermogenesis and thus athletic performance [7].

## 3. Resistance (R) and Muscular Performance Training and PEDs

Resistance training (R) is a vital component of every athlete's training regimen given its role in athletic performance. Thus, identifying nutritional strategies that enhance muscle strength, power, and function are essential (Table 4).

### 3.1. Creatine

Creatine, a component of phosphocreatine, is critical for rapid production of adenosine triphosphate (ATP) [27]. Along with creatine being the most well-researched sports supplement, it has been shown to enhance lean muscle mass, strength, and anaerobic performance and may also improve aerobic endurance [28]. Thus, there is strong evidence it is a potent performance enhancing nutrient.

*Mechanisms*. Creatine supplementation clearly increases intramuscular creatine and phosphocreatine concentrations [29–33]. Based on the role of phosphocreatine in energy production, this has commonly been proposed as an explanation for creatine's ergogenic effects [31, 34–36]. While one study found creatine to enhance phosphocreatine resynthesis [37], others have not, but have shown the higher phosphocreatine levels to persist throughout contraction and recovery [31, 38, 39]. As such, initial levels of phosphocreatine appear to be more important than its rate of resynthesis.

Protons are consumed when ATP is resynthesized from phosphocreatine [27], which implies that creatine may enhance performance by buffering against intracellular acidosis during exercise [35, 40]. Creatine may also act as a buffer by reducing reliance on glycolysis and the adenylate kinase reaction [35].

Creatine is known to increase intracellular fluid volume [41], which may increase glycogen [42] and protein [43] synthesis, and has been proposed as a mechanism of performance enhancement [41, 44]. However, investigation of creatine's influence on protein synthesis has led to conflicting results in both animals [45, 46] and humans [47–50]. Alternatively, creatine may indirectly increase protein synthesis by facilitating greater training volume [44].

Other possible mechanisms include increased energy efficiency of muscle contraction resulting from a faster relaxation response [51] and enhanced forced production from increased antioxidant capacity [52].

*Evidence*. In a large meta-analysis, creatine supplementation was found to increase either body weight or lean body mass in 43 of 67 trials [53]. Furthermore, our laboratory has shown creatine supplementation to be effective for increasing lean body mass, particularly when combined with resistance training [34].

Although the influence of creatine supplementation on lean body mass has not received much recent attention, several studies have further supported its benefit. In male professional soccer players, 5 days of creatine loading at 20 g·d^−1^ during typical training and competition led to increases in body mass and jumping power that did not occur with the placebo [54]. Two other recent trials, which did not control for creatine intake, provide some practical insights for using creatine to increase lean body mass. In male recreational bodybuilders, 4 weeks of creatine supplementation at 5 g·d^−1^, combined with resistance training, led to increases in lean body mass and 1 repetition maximum (RM) bench press with indication of greater benefit from postexercise versus preexercise supplementation [55]. The second trial also focused on recreationally trained men but included a creatine loading phase and lasted for 8 weeks [56]. Furthermore, for one of the groups, the rest interval between resistance exercises was progressively decreased by 15 seconds each week, which resulted in a lower training volume [56]. Despite the reduced training volume, increases in muscle cross sectional area for the upper arm and thigh, as well as 1 RM for the squat and bench press, were not different between groups, suggesting that creatine supplementation can be used to increase training efficiency [56]. Other recent trials have shown creatine to reduce postexercise levels of inflammation [57] and muscle damage [58], suggesting it may facilitate recovery.

*Practical Use*. A common dosage regimen for creatine is 20 g·d^−1^ during the first 4–7 days, followed by 5 g·d^−1^ thereafter [59]. As little as 2 g·d^−1^ has fully [30] or partially [32] maintained the intramuscular creatine levels achieved with loading, and 3 g·d^−1^ for 28 days has produced comparable levels without loading [30]. However, as a part of the PRISE protocol, an acute dosage of 2–5 g 1 hour prior to an R exercise bout may enhance muscular and physical performance. It is unclear if creatine intake from food will provide the same benefits as supplementation. However, herring, salmon, pork, beef, and cod are prominent sources containing 3–10 g·kg^−1^ [60, 61]. Chicken and rabbit are also within this range [62]. Therefore, it is possible to achieve a maintenance dose with whole foods [61], but a loading dose would be much less practical. For example, beef contains approximately 4.5 g·kg^−1^ of creatine [60], which translates to 0.8 g in a single 6 oz. serving.

### 3.2. Branched-Chain Amino Acids

The branched-chain amino acids (BCAAs), which include leucine, isoleucine, and valine, are essential nutrients involved in muscle protein synthesis and energy metabolism [63]. Leucine is particularly important for stimulating muscle protein synthesis [25], but BCAAs can be used collectively to enhance endurance, reduce muscle breakdown, and stimulate recovery after exercise.

*Mechanisms*. During exercise, BCAAs are catabolized into succinyl-CoA and acetyl-CoA, both of which can enter the citric acid cycle to support ATP resynthesis [63, 64]. This pathway has a critical role in exercise tolerance [65] and is likely fed by muscle protein breakdown, which can be reduced with BCAA supplementation [66]. Therefore, BCAAs can preserve muscle protein by acting as an energy substrate. Furthermore, BCAAs may enhance exercise performance by reducing central fatigue [67, 68] and enhancing fat oxidation [69–72].

Protein synthesis is the most well-known and arguably the most important mechanism through which BCAAs enhance performance. Although all three of the BCAAs contribute to protein synthesis, leucine is particularly important. This is because leucine activates translation initiation factors and the mammalian target of rapamycin (mTOR), which are influential in the regulation of protein synthesis [73–76].

*Evidence*. Some trials have shown BCAAs to enhance exercise capacity [72, 77–79] while others have not [80–85]. In a recent trial including 19 untrained males and 8 weeks of resistance training, 9 g·d^−1^ of BCAAs failed to change body composition or improve strength or muscular endurance to a greater extent than the placebo [86]. However, in a similar trial including 26 untrained men and 12 weeks of resistance training, 4 g·d^−1^ of leucine led to greater strength gains [87]. These contrasting results suggest that either leucine alone is more effective, or that 8 weeks is too short of a training period.

Further supporting the importance of leucine, a recent crossover trial including 9 military personnel found that increasing the leucine content of a 10 g essential amino acid (EAA) dose from 1.87 to 3.5 g led to greater muscle protein synthesis and less total-body protein breakdown following 60 minutes of cycle ergometry [88]. Similarly, another recent trial assessed myofibrillar protein synthesis following a bout of resistance exercise and found that increasing the leucine content of 6.25 g of whey protein from 3 to 5 g resulted in the same rate of protein synthesis as 25 g of whey [24]. However, the inclusion of additional BCAAs prevented this outcome, possibly due to increased competition for absorption [24].

*Practical Use*. As little as 77 mg·kg^−1^ of BCAAs has been shown to reduce muscle protein breakdown during exercise [66]. For EAAs, although 6 g has been shown to enhance protein synthesis [89], 10 g appears to be the optimal dose [26, 90].

While it is generally ideal to consume protein from whole-food sources, EAA supplementation has been suggested as an efficient method of promoting muscle growth while limiting caloric intake [91]. This is particularly relevant to athletes who need to lose or maintain weight. Furthermore, because exercising with a full stomach is generally not desirable [92], supplementation may be more appropriate for preexercise consumption.

A single acute serving of high-quality protein containing the optimal 10 g dose of EAAs contains approximately 1.8 g of leucine [93]. Relative to common protein sources, the leucine content of a 100 g (3.5 oz.) serving of beef, pork, chicken, turkey, salmon, cod, or tuna ranges from approximately 1.3 to 2.3 g [94]. Two eggs or a 100 g serving of haddock, shrimp, or scallops contains slightly less leucine, but still more than 1 g [94].

Finally, liquid sources of protein are known to elevate BCAA, EAA, and leucine concentrations more rapidly [95, 96], which can result in greater protein synthesis [97–99]. Whey [98] and milk [100], if well tolerated, are particularly effective.

## 4. PEDs for Interval (I) Sprint Training

A growing body of research has documented the benefits of interval sprint training (I) for improved anaerobic and aerobic athletic performance (Table 4). Certain nutritional strategies have proven effective to counter the increased acidic environment induced by I training and thus prolong training time and adaptations, all of which may directly enhance athletic performance.

### 4.1. Beta-Alanine

Beta-alanine is the rate-limiting precursor in the synthesis of carnosine, a cytoplasmic dipeptide that buffers intracellular H^+^ [101]. As such, it may reduce the acidic environment inside the muscle allowing for continued high-intensity anaerobic work performance and therefore may be suitable prior to and during an I exercise session.

*Mechanism*. Carnosine's role as an H^+^ buffer in the muscle is the first line of defense against local changes in pH. The absence of carnosine in isolated muscles leads to acidification and fatigue [101]. Therefore, the use of *β*-alanine supplementation to buffer H^+^ during high intensity exercise that causes muscle acidosis may extend the onset to fatigue by elevating intracellular carnosine concentrations [101] leading to increased work performance.

*Evidence*. Research has shown improved performance following *β*-alanine supplementation among different exercise modalities, such as swimming [102], cycling [103], running [104], and sprint performance following long endurance cycling [105]. However, these results are conflicting in nature with others reporting little or no change in performance [106–109] despite elevated carnosine concentrations [107, 110] or resistance to fatigue [107, 108]. Derave et al. [107] reported that four weeks of *β*-alanine supplementation (4.8 g·d^−1^) versus placebo in trained male athletes showed significant improvements in both dynamic knee extension torque (during the fourth and fifth bouts) and carnosine content in the soleus (47%) and gastrocnemius (37%). However, there were no differences in isometric strength or 400 m race time between groups. These findings contradict Ducker et al. [104] who found that male recreational runners improved 800 m race time following 4 weeks of *β*-alanine supplementation (6.4 g·d^−1^) versus placebo. Such contrasting results suggest that differences in training status may limit the effectiveness of *β*-alanine on improved performance more so than the dosage of *β*-alanine supplementation.

It is speculated that the effectiveness of *β*-alanine supplementation may be blunted in trained athletes due to the already elevated muscle buffering capacity from intense exercise training [111]. To compare the effects of *β*-alanine supplementation and training status, de Salles Painelli et al. [112] tested the effects of *β*-alanine supplementation in trained and nontrained cyclists. Forty males were separated in two groups based on training status (*N* = 20 endurance trained (T); *N* = 20 nontrained (NT) cyclists). Participants performed four 30 s lower-body Wingate bouts separated by 3 min, both before and after 4 weeks of either placebo or *β*-alanine supplementation (6.4 g·day^−1^). The sum of the four bouts represented the total work done (TWD) and the mean power (MPO) and peak power (PPO) output were obtained from each of the four bouts individually. *β*-Alanine supplementation was shown to significantly increase TWD in both T and NT groups with no significant difference in the T cyclist placebo group. Furthermore, it was found that MPO significantly improved in the T group during bouts 1, 2, and 4 but also improved in bout 4 for the NT group. It was concluded by de Salles Painelli et al. [112] that, despite training status, *β*-alanine improved both TWD and MPO during high-intensity exercise.

*Practical Use*. Research has found that *β*-alanine supplementation of 3–6 g·d^−1^ (~40–80 mg·kg^−1^·BW·d^−1^) for at least 4 weeks or longer will increase intramuscular concentrations (30–80%) possibly improving muscle buffering capacity [107, 113, 114]. However, a higher intake (~6 g·d^−1^) for four weeks has been shown to elicit greater carnosine concentrations and improvements in performance [103, 104, 112]. A single acute dosage prior to an I exercise session may elicit similar favorable buffering capacity.

### 4.2. Caffeine

Caffeine is the most widely consumed drug in the world and one of the most extensively studied ergogenic aids. It is well known for enhancing endurance [61, 115–118] but has also been shown to improve strength, power, and other aspects of high-intensity exercise [115, 117, 118]. Its effects are acute and peak with 30–60 minutes.

*Mechanisms*. The performance benefits of caffeine are related to enhanced fat oxidation and glycogen sparing. Caffeine is known to increase energy expenditure and fat oxidation, mostly through sympathetic nervous system activity (SNSa) [119], and other related mechanisms [61, 118, 120]. Inhibition of adenosine receptor activity, resulting from the molecular similarity between caffeine and adenosine, is the primary mechanism [116, 118, 121]. By binding to its receptors, adenosine can promote an increase in perceived pain and a reduction in arousal [116]. Exercise can accentuate this effect through the catabolism of ATP, adenosine diphosphate (ADP), and adenosine monophosphate (AMP) [116]. Therefore, caffeine-induced impairment of adenosine receptor activity may enhance performance by reducing the perception of discomfort and maintaining or enhancing motor unit firing rates [116]. Further supporting the role of pain perception, caffeine has been observed to increase concentrations of *β*-endorphins during exercise [122].

Peripheral mechanisms are also believed to contribute to caffeine's ergogenic effects [116, 117, 121]. The most notable is enhanced excitation-contraction coupling, resulting from increased potassium transport in muscle by Na^+^/K^+^ ATPase and increased release of calcium from the sarcoplasmic reticulum [116, 121].

*Evidence*. In a meta-analysis including 40 trials, caffeine had mild benefit for high-intensity exercise of short duration and stronger benefit for endurance exercise, but no benefit for graded exercise to exhaustion [123]. In a systematic review of 21 studies involving time trials of at least 5 minutes, caffeine enhanced performance by 2.3–4.3% [124]. For activities lasting 5 minutes or less, another systematic review found caffeine to improve intermittent exercise performance in 11 of 17 trials and strength-related measures in 6 of 11 trials [125]. Finally, in a meta-analysis including 27 trials for strength and 23 trials for muscular endurance, caffeine produced small but significant benefits for each attribute [126].

Trials published since 2013 have shown caffeine to improve agility [127, 128], jump height [127, 129, 130] and power [131], sprint performance [131], and sport-specific performance [127, 130–132] in athletes involved in a variety of sports, including basketball [129], rugby [131, 132], soccer [130], volleyball [127], and several racket sports [128]. Other recent trials have found improvements in cycling power output [133] and isokinetic knee extension torque during resistance exercise [134].

Two recent trials evaluated the influence of caffeine on exercise performed in a glycogen depleted state. In the first, which was a crossover with 12 competitive cyclists, 3 mg·kg^−1^·BW of caffeine resulted in similar power output during high-intensity interval training (HIIT) compared to the placebo with normal glycogen levels, indicating that caffeine attenuates the performance decline caused by glycogen depletion [133]. In the second crossover trial, which included 7 amateur cyclists, 5 mg·kg^−1^·BW of caffeine led to better 4 km time trial performance compared to the placebo with normal glycogen levels [135]. However, the difference was not significant.

*Practical Use*. The dosage of caffeine most commonly shown to enhance performance with minimal side effects is 3–6 mg·kg^−1^ [61, 118, 124]. It is possible to consume such a dosage from coffee [61], but the evidence comparing the efficacy of coffee and caffeine is conflicting [136, 137]. This discrepancy may be a result of variability in other coffee constituents [137], suggesting that caffeine is likely to be more reliable.

Athletes who regularly consume caffeine may have a higher tolerance and experience less benefit [138]. Furthermore, cessation of caffeine usage can result in withdrawal symptoms including headaches and impaired performance [138]. Therefore, to maximize benefit, usage should be discontinued at least 7 days prior to an event with a gradual reduction spread over 3-4 days [138]. Finally, because caffeine has been shown to negate the performance benefits of creatine [139], there appears to be little value in using them together.

### 4.3. Creatine

The mechanisms and practical applications of creatine were previously discussed in relation to resistance (R) training for muscular development. In regard to high-intensity exercise performance, creatine is most commonly recognized for its effect on strength but has also shown potential for enhancing anaerobic endurance.

In a meta-analysis of 7 trials, including a total of 70 subjects, creatine supplementation with concomitant resistance training led to a 6.85 kg greater increase in 1–3 RM bench press [59]. Similarly, among 37 subjects from 4 trials, there was a 9.6 kg greater increase in 1 RM squat [59]. Despite this evidence of enhanced strength, a meta-analysis of 10 trials, including a total of 92 participants, found no improvement in cycling power output [59]. However, in a larger meta-analysis, performance improvements were reported in 45 of 61 trials for activities lasting 30 seconds or less, in 17 of 25 trials for activities lasting between 30 and 150 seconds, and in 9 of 18 trials for activities lasting longer than 150 seconds [53]. Effect sizes were significant, although modest, for all measures, and were indicative of diminishing performance benefit with increased exercise duration [53].

Recent evidence indicates that creatine supplementation can enhance performance independently of training. In a trial including 77 men, creatine improved vertical jump, 20-yard shuttle run, 3-cone drill, and bench press endurance despite the lack of a training intervention [140]. Similarly, in two other trials lacking a training intervention, 7 days of creatine supplementation improved mean power during two bouts of the Wingate protocol [141], and 6 days of supplementation showed a tendency for increased lactate threshold, power output, and time to fatigue during incremental cycling [142].

### 4.4. Sodium Bicarbonate

Bicarbonate is a prominent buffer in human physiology. Supplementation with sodium bicarbonate increases blood pH and bicarbonate concentration, is particularly effective for enhancing anaerobic capacity, and may also improve strength and endurance [143, 144].

*Mechanisms*. Although the mechanisms are not fully understood, intramuscular acidosis has reduced muscle contractile capacity in multiple studies [145]. When exercise creates a demand for ATP that exceeds mitochondrial capacity, accumulation of protons released from glycolysis and ATP hydrolysis promote acidosis [146]. Although intramuscular acidosis has been argued to have a minimal effect on performance [147], sodium bicarbonate is known to increase pH and bicarbonate concentration [148, 149], which has persisted as the most likely mechanism of performance enhancement [148]. During exercise, sodium bicarbonate has been shown to result in higher lactate levels during exercise despite a similar intramuscular pH [150] and promotes greater glycogen and phosphocreatine utilization [151], suggesting increased capacity for anaerobic energy production. Furthermore, exercise-induced acidosis can inhibit oxidative phosphorylation [152], which implies that the buffering effect of sodium bicarbonate may enhance aerobic energy production as well.

Sodium bicarbonate supplementation has led to greater muscle contraction velocity following 50 minutes of high-intensity cycling [153], suggesting it may reduce neuromuscular fatigue in addition to enhancing energy production.

*Evidence*. In a meta-analysis including 29 trials, sodium bicarbonate was found to increase anaerobic exercise capacity, with the largest improvements observed for time to exhaustion [149]. The greatest benefit was observed in conjunction with larger drops in pH during exercise [149], suggesting that the benefits are most applicable to glycolytic activities. A more recent meta-analysis, including 38 studies, standardized all results as a measure of mean power production during time trial performance and found a clear but modest performance benefit [148]. Improvement increased slightly as exercise duration increased beyond 1 minute, but durations beyond 10 minutes slightly reduced benefit [148].

In a recent trial including 11 well-trained endurance athletes, 0.3 g·kg^−1^·BW of sodium bicarbonate was consumed prior to exercise for 5 consecutive days [154]. A similar improvement in time to exhaustion was maintained each day [154], suggesting that supplementation is appropriate for multiday events. Two other recent trials evaluated sodium bicarbonate in combination with beta-alanine. A single 0.3 g·kg^−1^·BW dose of sodium bicarbonate improved repeated sprint performance in team-sport athletes [155] and improved sprint swimming performance in competitive male swimmers [156]. In both trials, however, the addition of beta-alanine failed to further enhance performance.

In another recent trial including well-trained rowers, preexercise consumption of 0.3 g·kg^−1^·BW of sodium bicarbonate throughout 4 weeks of HIIT failed to improve time trial performance compared to the placebo [157], suggesting that supplementation may not be effective for enhancing training adaptations. However, this is in contrast to a previous trial that observed greater improvements in lactate threshold and time to exhaustion [158].

*Practical Use*. The dose of sodium bicarbonate most frequently associated with performance enhancement is 0.3 g·kg^−1^ [148, 149]. However, benefits have been observed with as little as 0.15 g·kg^−1^·BW [159]. Common gastrointestinal symptoms can be avoided during competition by consuming the dose 3 hours prior to initiating I exercise [160]. Consuming the dose with food may also help [161] but increases the importance of distancing intake from the start of competition [92]. Alternatively, smaller doses can be consumed over several days preceding an I event [162].

Although the buffering effect of alkalizing food [163, 164] is unlikely to produce the 0.05 increase in pH or 6 mmol·L^−1^ increase in bicarbonate that appear necessary for performance improvement [161], potential for benefit may still exist. The alkalizing potential of food is primarily attributed to potassium salts, which increase bicarbonate availability when metabolized [165–167]. Vegetables and fruit are most abundant in potassium salts [165] and have the highest alkalizing potential [168]. Therefore, in conjunction with the numerous health benefits of vegetables and fruit [169], as well as potassium [170], high intakes may facilitate performance enhancement. Furthermore, the alkalizing potential of vegetables and fruit can help to offset the acidifying effect of protein [165, 167, 168], which athletes require in greater amounts [92]. In support of this, a high vegetable intake has recently been shown to increase capillary pH in adults during rest and submaximal exercise [171]. Bicarbonate can be more directly incorporated into the diet with mineral water [163, 172] or baking soda.

## 5. PEDs for Stretching (S) and Restorative Training

It is well known that intense exercise training induces muscle damage, including an imbalanced ratio of protein breakdown to protein synthesis and increased muscle soreness (i.e., perception of pain) and inflammation [173]. A growing number athletes turn to common nonsteroidal anti-inflammatory drugs (NSAIDs) (i.e., ibuprofen) to alleviate or reduce the perception of pain and to attenuate the inflammatory response [174]. Furthermore, many athletes will perform certain modes of S exercise (i.e., yoga, stretching, and massage) as a form of restorative training to actively alleviate pain from previous strenuous exercise [175]. More recently, the combination of both active recovery exercises and nutraceuticals in the form of BCAA [176], ginger [177], turmeric [178], omega-3 (PUFAs) [179], and tart cherry [180, 181] have been suggested as natural alternatives for reducing exercise-induced inflammation (Table 4).

### 5.1. Ginger

Ginger (*Zingiber officinale*) is one of the ten most commonly used natural complementary and alternative medical treatments in the United States [182] and has been suggested as a possible alternative to pharmaceuticals for reducing pain and/or inflammation [177].

*Mechanism*. In animal models, ginger and its chemical constituents gingerols, shogaols, paradols, and zingerone are agonists to the transient receptor potential vanilloid subfamily, member 1 (TRPV1) that function in central and peripheral nociceptive signaling by inhibiting the release of prostaglandins and leukotrienes [183–185]. Ginger has been proposed as an effective analgesic based on its evidence as a natural medicinal in reducing pain and inflammation. Moreover, there are inconsistent findings from NSAIDs such as ibuprofen, naproxen, aspirin, and diclofenac as effective analgesics following eccentric exercise [186]. Thus, ginger consumption may be more efficacious for reducing exercise induced pain and inflammation through activation of TRPV1.

*Evidence*. It has been found that the use of ginger as pain treatment, with smaller dosages (30 to 510 mg·d^−1^) and longer durations (4 to 36 weeks), resulted in reductions in knee or hip pain in individuals with osteoarthritis. Black et al. [177] reported that following eccentric exercise (18 eccentric elbow flexor contractions at 120% of 1 RM) 2 g of both raw and heat-treated ginger for 11 days significantly decreased the perception of pain following exercise. Evidence supports the use of ginger to aid recovery from muscle-damaging exercise and for longer durations of intake (>2 days), as a single-acute dose had no effect on pain perception following low-moderate (60% VO_2 peak_) intensity cycling [187]. Thus, effectiveness of ginger on pain perception may prove beneficial as treatment for alleviating intense, muscle-damaging (i.e., eccentric) exercise induced pain, more so as an alternative to pharmaceuticals.

*Practical Uses*. A higher dosage of 6 g of ginger may lead to possible stomach irritation and therefore a lower dose of 2–3 g of ginger is suggested as it has been shown to be effective in reducing both pain following exercise and blood sugar concentrations [177]. This dosing regimen also allows for daily consumption of ginger beyond just on S days and is well-tolerated.

### 5.2. Curcumin

Curcumin, a polyphenol responsible for the yellow color of turmeric (curry powder), is known to reduce inflammation and influence metabolic function [188]. As such, curcumin has the potential to support recovery and performance on S training days by promoting metabolic health.

*Mechanism*. Curcumin is known to regulate inflammation and directly interact with adipocytes, pancreatic cells, and muscle cells [188]. Curcumin has been well documented to regulate biochemical and molecular pathways by modulating molecular targets such as transcription factors, cytokines, enzymes, and the genes responsible for both cell proliferation and apoptosis [189].

*Evidence*. Curcumin has recently been shown to reduce pain associated with delayed onset muscle soreness (DOMS) following downhill running [190]. Chuengsamarn et al. [178] tested the effects of 250 mg of curcumin compared to placebo (corn starch) ingested twice a day for 6 months on atherogenic risks in individuals with type II diabetes mellitus (T2DM). After 6 months of supplementation it was found that curcumin significantly decreased pulse wave velocity, increased adiponectin and decreased leptin, and also decreased homeostasis model assessment-estimated insulin resistance (HOMA-IR), triglycerides, uric acid, and abdominal obesity (visceral fat and total body fat). These findings indicate that daily incorporation of curcumin will significantly alter the proinflammatory cytokine leptin and the anti-inflammatory cytokine adiponectin, as well as reduce abdominal obesity, all of which helps to ameliorate the atherogenic risks of T2DM individuals [178].

Though a plethora of information on the positive effects of curcumin on diseased individuals has been well documented [189], only one study known to date has specifically reviewed the effects of curcumin on oxidative stress following exercise in humans. Takahashi et al. [191] tested the effects of curcumin on oxidative stress and antioxidant capacity following exercise (60 min at 75% of VO_2max⁡_) in ten healthy men. The participants completed three trials in a random order of ingesting either placebo, 90 mg of curcumin-single (before exercise only, 2 hr), or 90 mg of curcumin-double (before and immediately after exercise). It was found that immediately following exercise, both the curcumin-single and double groups had significantly lower derivatives of reactive oxygen metabolites and plasma thioredoxin-1 and significantly elevated biological antioxidant potential and reduced glutathione concentrations compared to the placebo group. These results suggest that exercise-induced oxidative stress may be attenuated by increasing blood antioxidant capacity from curcumin supplementation [191].

*Practical Use*. Though the consumption of curcumin has shown to be safe and has been consumed by ancient people for thousands of years the scientific analysis and understanding of curcumins effects are still being researched. It has been noted that when working with certain diseased populations or those unaccustomed to curcumin lower dosages (<250 mg) have been shown to reduce abdominal fullness or pain. Dosages of 90–250 mg daily, particularly on S training days, may be an effective adjuvant therapy to aid recovery and healing from strenuous exercise. A possible limitation is the relatively low bioavailability of curcumin consumed orally. However, there have been recent modifications in producing a bioavailable and higher orally absorptive curcumin known as Theracurmin [192].

### 5.3. Omega-3 Poly-Unsaturated Fatty Acids (PUFAs)

The main components of omega-3 polyunsaturated fatty acids (PUFAs) found in fish oil are eicosapentaenoic acid (EPA) and docosahexaenoic acid (DHA) and are produced from the omega-3 fatty acid alpha-linolenic acid (ALA).

*Mechanism*. Because EPA and DHA are not naturally synthesized in the body and the breakdown of ALA to produce EPA and DHA is enzymatically inefficient, the consumption of fish oil through diet or supplementation is important for providing adequate EPA and DHA concentrations. Both EPA and DHA are eicosanoids that have anti-inflammatory, antithrombotic, antiarrhythmic, and vasodilatory properties. The derivative of the longer chain fatty acid linoleic acid (LA) is arachidonic acid, the precursor to the proinflammatory and prothrombotic eicosanoids. Because ALA and LA compete for the same enzymes in the production of the longer chain fatty acids EPA and arachidonic acid, the consumption of fish or fish oil avoids the enzymatic competition to convert ALA to EPA by providing EPA and DHA directly [193].

*Evidence*. More commonly known for their cardiovascular benefits, EPA and DHA have been documented to reduce inflammation, as well as delayed onset muscle soreness (DOMS) or the perception of pain from exercise [179, 194–196]. When supplementing with EPA and DHA either prior to or during exercise, or the combination of both, research has found decreased resting levels of inflammatory biomarkers (2,224 : 2,208 mg·d^−1^, 6 wks) [194], decreased acute-phase proteins after exercise (1.75 : 1.05 g·d^−1^, 3 wks) [179], and improved perceived muscle soreness, pain, and range of motion 48 hrs post exercise (324 : 216 mg·d^−1^, 30 days and 48 hrs during recovery) [195]. More recently, Jouris et al. [196] reported the attenuation of DOMS when consuming EPA and DHA at a 2 : 1 ratio (2,000 : 1,000 mg·d^−1^) for 7 days following an eccentric arm-curl exercise protocol. Yet, despite these beneficial findings, there have been reports of little or no change in inflammation or DOMS following exercise [197, 198]. Recently, in addition to ameliorating pain and inflammation, supplementation with omega-3 PUFA for 8 weeks (1.86 : 1.50 g·d^−1^ EPA : DHA) was shown to augment the activation of the mTOR-p70s6k signaling pathway stimulating protein synthesis in older adults [199]. Thus, omega-3 supplementation may also prove beneficial for the prevention or management of sarcopenia or the atrophy of skeletal muscle [199].

*Practical Use*. It should be noted that fish oil consumption at higher levels (>4 g per day) may increase the risk of bleeding from decreased adherence of blood platelets and lower blood pressure. Hence, individuals with already low blood pressure or increased risk of hemorrhage should consume moderate to lower intakes of omega-3 PUFA. Athletes that wish to mitigate the effects of exercise-induced inflammation and DOMS are suggested to incorporate omega-3 FA in their diet, especially during S days, and are suggested to do so with 1-2 g·d^−1^ of an EPA : DHA ratio of 2 : 1 [200], or 2–4 g·d^−1^ for those with higher blood lipid profiles or rheumatoid arthritis [193]. A designated safe and general consumption dose of omega-3 PUFA (EPA + DHA) for athletes to consume is ≤3,000 mg·d^−1^ (3 g), as recommended by the US Food and Drug Administration [201]. For many individuals omega-3 capsule supplementation is convenient for ensuring adequate consumption of PUFA, and an alternative for vegetarians, but for those who are able to incorporate whole food sources, flax seeds, walnuts, sardines, and salmon are considered excellent sources of rich omega-3 (e.g., EPA, DHA, and ALA). Because of the concern of high levels of mercury the following fishes have been given as examples of 1 g servings of EPA : DHA because of their low mercury content: 4.0 oz. Tuna (Canned, Light), 2.0–3.5 oz. of salmon (Atlantic, wild), 15 oz. of catfish, and 11 oz. of shrimp (mixed species). For more recommendations of grams of EPA and DHA for various types of fishes and servings see the review by Covington [193].

### 5.4. Tart Cherry

Cherries are known to be a rich source of bioactive compounds with antioxidant and anti-inflammatory effects [202, 203]. Both the antioxidant and anti-inflammatory effects of cherries are believed to contribute to their potential to reduce pain and enhance exercise recovery [202].

*Mechanisms*. Although the precise mechanisms of how cherry consumption influences exercise recovery are not fully understood, the mechanical muscle damage induced by eccentric contraction is unlikely to be affected [202]. Instead, improvements in recovery are most likely related to the attenuation of secondary oxidative stress and inflammation [202]. The anthocyanins from both sweet and tart cherries are known to inhibit cyclooxygenase-I and cyclooxygenase-II [204], which provides at least a partial explanation for their anti-inflammatory effects. Tart cherries have a more potent effect.

*Evidence*. Although the influence of tart cherry on exercise recovery has only been investigated to a limited extent, the available evidence is very promising. Connolly et al. [180] assessed the effect of tart cherry juice (TCJ) on recovery from maximal elbow flexion contractions. The trial included 14 men who consumed 12 oz. of tart cherry juice twice per day for 8 days. Eccentric contractions were performed on the 4th day and recovery was assessed during the subsequent 4 days. The TCJ significantly reduced loss of strength and pain during recovery [180]. However, no differences were observed in tenderness or loss of range of motion [180]. A similar trial was conducted to determine if the response to tart cherry juice differed for well-trained athletes. In this trial, TCJ was administered as TCJ concentrate of 30 mL (1 oz.) twice per day for 7 days before and 2 days after knee extension exercise performed at 80% of maximum voluntary contraction (MVC) [205]. Consistent with the previous trial, TCJ significantly reduced loss of strength during the two days of recovery, but without any differences in muscle tenderness [205]. The TCJ also reduced protein carbonyl levels during recovery, suggesting a reduction in oxidative stress [205]. These data support TCJ as an effective PED aid following intense, muscle damaging R exercise as a result of mitigating the subsequent oxidative damage [205].

Several trials have also focused on recovery from endurance (E) exercise. Howatson et al. [206] evaluated the effect of two 8 oz. servings per day of TCJ supplementation for 5 days prior, the day of, and 2 days following a marathon run. The tart cherry juice resulted in significantly faster recovery of isometric strength, reduced inflammation and oxidative stress, and increased antioxidant capacity during the subsequent 2 day recovery [206]. In another running trial, consumption of tart cherry juice for 7 days prior to and during a 26.3 km relay race significantly reduced perceived pain following the race [207].

*Practical Use*. Consumption of approximately 45 sweet Bing cherries per day has been shown to reduce markers of inflammation [208, 209]; however, it is not clear whether the antioxidant and anti-inflammatory potentials of sweet cherries are comparable to tart cherries. Furthermore, most of the available evidence indicating a benefit from tart cherries is based on consumption of juice containing the equivalent of 90–120 cherries per day or 12–16 oz [180, 205–207]. As such, practicality and the limited scope of available evidence favor the use of tart cherry juice. The TCJ used in the aforementioned studies was derived directly from fresh cherries in concentrate or juice form making it feasible for most people to consume.

Emerging evidence indicates that oxidative stress is an important signaling mechanism for muscle remodeling [210] and may therefore be necessary for beneficial adaptations to exercise [211]. This concern is supported by evidence of antioxidant supplementation inhibiting adaptation to exercise [211]. Furthermore, anti-inflammatory substances such as nonsteroidal anti-inflammatory drugs present a similar concern. Similar to antioxidant containing foods, such as TCJ, NSAIDs reduce inflammation by inhibiting cyclooxygenase activity. There is indication of this mechanism inhibiting regeneration of muscle [212] and connective tissue [213], which could impair adaptations to exercise [212] and increase injury risk [213]. As such, the long-term use of antioxidants and NSAIDs may be contraindicated for athletes pursuing enhanced muscle mass development. Based on these findings, acute supplementation with TCJ may be most effective endurance sessions or competitions, rather than for continual use.

## 6. PEDs for Endurance (E) and Aerobic Training

More athletes are choosing nutritional supplements, from both natural and organic sources, to gain a competitive advantage in endurance-based sports. The increased energy demands of endurance activities require fluid, electrolyte, and energy consumption during training and competition (Table 4). Facilitating the delivery of these key nutrients to working muscles is paramount to athletic performance.

### 6.1. Beet Root Juice (BRJ)

Beetroot juice (BRJ) is among the most popular nutritional supplements to improve endurance performance [214]. Much of this is due to an increased consumption of organic and natural foods [215]. Thus, the trend for organic and natural food products is particularly relevant for athletes at all levels of competition. BRJ is particularly popular among endurance (E) athletes, because of its high concentration of nitrate that has been hypothesized to enhance endurance. For example, there is both anecdotal and scientific support for BRJ to improve time-trial endurance [216] and time to exhaustion [217], reduce steady-state oxygen consumption [218], and increase peak power and work rate at the gas exchange threshold [219].

*Mechanisms*. Several mechanisms have been postulated for the endurance exercise improvement effects of BRJ. A reduction in phosphocreatine (PCr) degradation and the reduction of build-up of ADP and inorganic phosphate (Pi) at the same relative exercise intensity following BRJ consumption [216, 219] are likely mechanisms responsible for the decrease in O_2_ cost (oxidative phosphorylation) of exercise and increased time to exercise failure (reduced muscle fatigue). Beetroot has a high nitrate (NO_3_^−^) content (>250 mg/100 g of fresh weight), among the highest assessed, and contains more than other foods high in NO_3_^−^ including spinach, celery, arugula, and carrot juice [220]. Nitrate is reduced to nitrite via bacteria in the oral cavity and by specific enzymes (e.g., xanthine oxidase) within tissues. There are several pathways to metabolize nitrite to nitric oxide (NO) and other biologically active nitrogen oxides [221]. Nitric oxide is a signaling molecule formed in the endothelium by the enzyme endothelium nitric oxide synthase (eNOS) which triggers the vasculature to relax (vasodilatation) by interacting with vascular smooth muscle leading to increased blood flow [222] at rest [223] and during exercise [224].

Given these properties, NO has gained a lot of attention for possible E exercise improvements including increased O_2_, glucose, and other nutrient uptake to better fuel working muscles. Currently there is no means to provide NO supplementation through the diet (as it is a gas), thus BRJ and its high nitrate concentration are used as a means to generate NO endogenously. Indeed, there is an impressive and growing body of scientific data in support of whole food sources of inorganic nitrate, such as that found in BRJ, showing improved athletic performance.

*Evidence*. While there is very limited scientific data demonstrating BRJ's effect on resistance (R) exercise [225], the vast majority of data strongly supports its beneficial effect on improving E performance. Lansley et al. [217] recruited 9 healthy, physically active men who consumed either 0.5 liters of BRJ (6.2 mmol·d^−1^ of NO_3_^−^) or 0.5 liters of NO_3_^−^-depleted BRJ placebo (0.0034 mmol·d^−1^ of NO_3_^−^) for 6 days followed by acute bouts of submaximal and high-intensity (to exhaustion) running and incremental knee-extension exercises. BRJ consumption increased plasma nitrite by 105% and reduced the O_2_ cost for constant-work-rate moderate and severe-intensity running by ~7% compared to placebo. In addition, time to exhaustion was increased during severe-intensity running by ~15% and incremental knee-extension exercise by ~5% with BRJ compared to placebo. These findings suggest that performance benefits (oxygen sparing and enhanced exercise tolerance) of consuming BRJ are attributed to its high NO_3_^−^ content. More recently, Murphy et al. [226], using a double-blind placebo-controlled crossover trial, had 11 recreationally fit men and women consume either baked beetroot (200 g with ≥500 mg NO_3_^−^) or an isocaloric placebo (cranberry relish) 75 minutes prior to performing a 5 km time trial treadmill run to determine whether whole beetroot consumption would improve running performance. They observed a nonsignificant, 41-second faster finishing time (12.3 ± 2.7 versus 11.9 ± 2.6 km·h^−1^, resp.; *P* = 0.06) following beetroot consumption compared to placebo. Most impressive, during the last 1.1 miles (1.8 km) of the 5 km run, running velocity was 5% faster (12.7 ± 3.0 versus 12.1 ± 2.8 km·h^−1^, resp.; *P* = 0.02) and rating of perceived exertion was lower (13.0 ± 2.1 versus 13.7 ± 1.9, resp.; *P* = 0.04) during the beetroot trial compared to the placebo. Thus, it appears that the ingestion of whole-foods containing inorganic NO_3_^−^ (such as beetroot or BRJ) increase plasma nitrite and ultimately NO levels which favorably affect the cellular and vasculature pathways which likely result in the observed improvements in endurance athletic performance.

Given the favorable impact of BRJ on E performance, it would seem likely that BRJ would also favorably impact other markers of athletic performance. As such, Lansley et al. [216] examined the effects of BRJ ingestion on power output, oxygen consumption (VO_2_), and performance cycling time trials (TT) using nine competitive male cyclists who consumed either 0.5 liters BRJ (6.2 mmol of NO_3_^−^) or placebo containing nitrate-depleted BRJ (0.0047 mmol of NO_3_^−^) before each TT of 4 or 16 km. BRJ consumption increased plasma nitrite by 138% and resulted in significantly reduced time to completion and increased power output during both the 4 km (2.8% and 5%, resp.; *P* < 0.05) and 16 km TT (2.7% and 6%, resp.; *P* < 0.05) compared to the placebo treatment.

Similarly, Bailey et al. [218] supplemented eight healthy, recreationally active men with 0.5 liters of BRJ (5.5 mmol·d^−1^ of NO_3_^−^) or a low-calorie black currant juice cordial (negligible NO_3_^−^ content) for 6 days while performing moderate (80% gas exchange threshold) and intense cycling (70% of the difference between the power output at the gas exchange threshold and VO_2 peak_) protocols during the last 3 days. BRJ ingestion increased the average plasma nitrite by 96% and increased the time to task failure by ~16% during fixed high intensity exercise. The authors concluded that increased dietary inorganic NO_3_^−^ consumption from BRJ has the potential to improve high-intensity exercise tolerance.

These data confirm that BRJ improves endurance exercise performance; however, the minimal time needed to use BRJ for a performance benefit remains to be elucidated. One attempt to answer this question was reported by Vanhatalo et al. [219] in which they examined the effects of acute (1 and 5 days) and chronic (15 days) BRJ consumption on a moderate-intensity exercise bout (90% gas exchange threshold) and an incremental cycle ergometer ramp test (increasing work rate by 1 W every 2 sec (30 W/min)) to exhaustion.

Eight healthy subjects (5 males, 3 females) consumed either 0.5 liters BRJ (5.2 mmol·d^−1^  NO_3_^−^) or a placebo (blackcurrant juice cordial with negligible NO_3_^−^ content) for 15 days and were exercise tested on days 1, 5, and 15. Plasma nitrite was significantly increased on all test days following BRJ compared to placebo. The O_2_ cost of moderate-intensity exercise (increase in VO_2_ relative to the increase in external work rate) was lower during BRJ and was maintained throughout the 15 days (*P* = 0.002). VO_2max⁡_, peak power output, and the work rate associated with the anaerobic threshold were all higher following 15 days of BRJ consumption compared to placebo and baseline conditions. In addition, BRJ systolic blood pressure was significantly lower at 2.5 hours after ingestion as well as 2, 12, and 15 days after ingestion compared to PL (−3%; *P* < 0.05). Diastolic blood pressure decreased with BRJ compared to PL (−5%; *P* < 0.01).

The authors concluded that acute (1–5 days) dietary NO_3_^−^ supplementation significantly decreased blood pressure and the O_2_ cost of submaximal exercise and increased VO_2max⁡_ and peak power output and these outcomes were maintained for at least 15 days with continued BRJ supplementation [219]. While most studies agree with these findings [227–229], others note that highly trained athletes (average VO_2max⁡_ of 72  ±  4 mL·kg^−1^·min^−1^) may not have the same response to BRJ [230], suggesting that the impact of BRJ may be influenced by the training status of the individual.

In nonathletic populations, the impact of BRJ also has a significant positive impact on endurance performance. Kenjale et al. [231] provided 8 patients with peripheral arterial disease either 0.5 liters of BRJ (18.1 mmol·L^−1^  NO_3_^−^) or an isocaloric placebo on two separate occasions while performing an incremental, graded treadmill running test and demonstrated an increased exercise tolerance (walked 18% longer before claudication pain onset and experienced a 17% longer peak walking time), and decreased fractional O_2_ extraction. These findings support BRJ to enhance peripheral tissue oxygenation in hypoxic areas and increase exercise tolerance in individuals with peripheral arterial disease. Thus, strong scientific evidence supports BRJ supplementation as an effective ergogenic aid for both athletes and nonathletes alike in order to improve endurance/aerobic exercise performance.

*Practical Use*. It is important to note that the acute dose of BRJ used in most research studies is approximately 0.5 liters or ~16 fl. oz. There are several ways to incorporate BRJ into an athlete's diet. One strategy is to prepare the BRJ from the whole beets using the following technique: remove the stalks and thoroughly wash the beets, cut into cubes, submerge in water, bring to a boil and then simmer for 45 minutes until beets are tender, allow to cool, pour off the fluid, and place in refrigerator (lasts up to 5 days) or freeze (up to 3 months). Consume 16 fl. oz. alone or mixed with another antioxidant-rich juice (tart cherry, grape, cranberry, and pomegranate) on endurance exercise days. Another technique is to thoroughly blend 2-3 whole beets (stalks removed) in a food processor, blender, or juice compressor.

### 6.2. Caffeine

The mechanisms through which caffeine enhances performance, as well as the practical considerations for caffeine use, were previously discussed in relation to anaerobic performance. In addition to the potential for caffeine to enhance anaerobic performance, meta-analysis has indicated it is more effective for enhancing aerobic performance [123].

Several recent studies have demonstrated the beneficial influence of caffeine on endurance performance. In 10 well-trained cross-country skiers, 6 mg·kg^−1^·BW of caffeine consumed 45 minutes prior to exercise led to better performance and reduced rating of perceived exertion (RPE) during an 8 km double-poling time trial [232]. Similarly, in trained cyclists, 200 mg of caffeine consumed 60 minutes before exercise improved 40 km time trial performance [233], and 5 mg·kg^−1^·BW of caffeine consumed 60 minutes before exercise, either in supplement form or from coffee, improved performance during an approximately 45 minute time trial with a target of 70% of maximal work output [137]. In addition, 3 mg·kg^−1^·BW of caffeine consumed 90 minutes prior to exercise has been shown to improve performance during an approximately 60 minute work-based time trial during hot conditions [234].

### 6.3. Carbohydrate and Fat Intake

Although protein contributes to energy production during E exercise, it is a small contribution relative to fat and carbohydrate [235]. As such, optimal macronutrient intake for supporting the energy demands of E athletic performance is primarily related to fat and carbohydrate.

Storage capacity for glycogen is greatly limited compared to fat and is therefore more tightly regulated [236]. Furthermore, reduced glycogen availability is commonly associated with fatigue [237–239], which implies that it may be advantageous to adjust macronutrient intake in a manner that either spares glycogen or reduces dependency on it. Two contrasting strategies for reducing dependency on glycogen are increasing carbohydrate intake to maintain high glucose and glycogen availability or restricting carbohydrate intake to promote adaptations that increase reliance on fat oxidation.

*Mechanisms*. Despite the well-established associations between glycogen depletion and fatigue, the mechanisms are not well understood [237–239]. However, it is clear that shifts in macronutrient intake alter the balance between fat and carbohydrate oxidation. In contrast to carbohydrate oxidation, which is largely influenced by carbohydrate intake, fat oxidation is influenced more so by carbohydrate intake than fat intake [236, 240]. More specifically, fat oxidation increases as carbohydrate intake decreases [236, 240].

Carbohydrate metabolism inhibits fat oxidation, and one mechanism for this has been eloquently isolated to the carnitine palmitoyltransferase (CPT) system, which transports long-chain fatty acids through the inner mitochondrial membrane. Infusion of glucose and insulin has been shown to inhibit oxidation of long-chain fatty acids, but not medium-chain fatty acids, implicating the CPT system as a location of inhibition [241]. Similar results have been observed in conjunction with elevated levels of malonyl CoA [242], which is known to inhibit the CPT system [243]. Furthermore, insulin activates acetyl CoA carboxylase (ACC), which catalyzes the production of malonyl CoA [244]. As such, carbohydrate intake is likely to inhibit fat oxidation by promoting insulin release, which then increases production of malonyl CoA by ACC and, in turn, inhibits the CPT system from transporting long-chain fatty acids into mitochondria [243, 245].

In addition to acute shifts in substrate selection, consistent changes in macronutrient intake can promote adaptations that may further enhance energy metabolism. For example, 5 days of reduced carbohydrate and increased fat intake in actively training cyclists increased genetic expression in skeletal muscle for fatty acid translocase (FAT), fatty acid binding protein, and *β*-hydroxyacyl CoA dehydrogenase (*β*-HAD), all of which are related to fat oxidation [246]. Similarly, 2 days of reduced carbohydrate and increased fat intake following glycogen depleting exercise led to increased expression for FAT and uncoupling protein 3 (UCP3), while a higher carbohydrate diet led to increased expression for glucose transporter type 4 and glycogenin [247]. In contrast, even without a change in regular diet, the consumption of glucose during moderate exercise has been shown to inhibit expression of FAT, UCP3, and CPT1 compared to the same exercise performed in a fasted state [248].

In addition to enzymatic changes, reduced carbohydrate and increased fat intake have been shown to increase intramuscular fat storage in conjunction with a lower respiratory quotient during exercise in both trained [249–251] and untrained [252] subjects. Although reduction of carbohydrate intake typically results in lower glycogen levels compared to a high-carbohydrate intake [251, 252], similar glycogen levels were maintained in one trial in conjunction with increased intramuscular fat and decreased respiratory quotient during exercise [250].

*Evidence*. It is well established that carbohydrate consumption prior to or during prolonged exercise enhances performance [238, 253–255]. In contrast, a number of trials have shown a high-fat and reduced-carbohydrate diet to increase fat oxidation or reduce reliance on glycogen during exercise, but with mixed effects on performance, ranging from deleterious to advantageous [250, 252, 256–267]. A meta-analysis of 38 trials found high-carbohydrate intake to be more beneficial, but the results were concluded to be unreliable due to heterogeneity [268]. Furthermore, benefit was minimal for trained subjects [268], and the results were skewed by intervention durations of less than 7 days, which may have not been enough time for adaptation to reduced carbohydrate intake [262, 268].

Given that carbohydrate restriction reduces glycogen [251, 259, 262, 263, 269, 270], limited capacity for high-intensity exercise is expected. However, the evidence is mixed [250, 257–259, 265, 266, 270–272], with indication that it is possible to maintain high-intensity exercise capacity even when carbohydrate is restricted to less than 10% of energy intake [259, 270–272]. Furthermore, normal glycogen levels and thus performance can be maintained with moderate carbohydrate restriction [250, 252], as well as with supplementation of carbohydrate during the exercise bout, if needed.

*Practical Use*. It is commonly recommended that athletes follow a high-carbohydrate diet to replenish glycogen and maintain blood glucose [92, 273]. However, a high-fat and reduced-carbohydrate diet may be an effective alternative [237, 274, 275]. The equivocal evidence indicates that a wide range in the ratio of fat and carbohydrate intakes can support high-level performance, although the enhancements in fat utilization observed with carbohydrate restriction are unlikely to have ergogenic value beyond that of a high-carbohydrate diet [276–278]. Despite this, fat adaptation can still be compatible with optimal performance and may have beneficial implications for weight management, training adaptation, and metabolic health. A meta-analysis of 87 trials [15] and a number of more recent trials [271, 279, 280] have shown carbohydrate restriction to have a more favorable influence on body composition during weight loss, particularly when combined with resistance training [281–283]. Furthermore, carbohydrate restriction has clearly been shown to have a highly favorable effect on cardiovascular and metabolic risk factors [284–289]. It has been suggested that only a quarter of the population can tolerate the current recommendations for carbohydrate intake without developing signs of metabolic dysfunction [275]. Although athletes are less susceptible [275], the presence of metabolic risk factors in athletes is not rare, especially in sports that favor a heavier body weight [290, 291]. As such, a high-fat and carbohydrate-restricted diet can be a valuable alternative for athletes who need to manage body weight or have signs of metabolic impairment. In addition, the lower glycogen levels that result from carbohydrate restriction may enhance adaptations to endurance training [273, 292, 293].

Overall, a high-carbohydrate intake is not the only way to support optimal endurance performance. However, carbohydrate intake is likely to be more important for high-intensity performance [278, 294]. Furthermore, there may be considerable variation in the optimal macronutrient ratio for each athlete [256, 262]. Given the equivocal evidence, athletes should determine their optimal ratio of fat and carbohydrate intakes based on a combination of factors including the demands of their sport, their individual response to different macronutrient ratios, and any concerns related to health or body composition. Considerations related to health and individuality are especially applicable to nonelite athletes, who have less reason to prioritize performance over wellbeing. One strategy to determine an ideal and individualized macronutrient ratio, which would be best implemented during the off-season, is to restrict carbohydrate for several weeks and then gradually increase carbohydrate intake until the minimal effective dose needed to sustain performance can be identified. This may include carbohydrate supplementation during exercise, if needed. However, it is important to understand that sufficient adaptation to carbohydrate restriction may take 2–4 weeks [262] and that further adaptation may continue beyond the 4th week [295]. Another approach is to restrict carbohydrate intake long enough to promote fat adaptation and then increase carbohydrate intake prior to or during a competition in order to restore glycogen levels. Enhanced fat oxidation has been shown to persist with such a strategy, although to a lesser extent, and with mixed effects on performance [296].

### 6.4. Fluid Hydration: Glycerol and Electrolytes

Fluid intake and adequate hydration are critical during E training sessions and competition events. Fluid intake helps to maintain hydration, body temperature (thermoregulations), and plasma volume. For events lasting longer than one hour, athletes need fluids containing carbohydrates and electrolytes rather than water alone. Reduction in body water, availability of carbohydrates, and an inadequate electrolyte balance during prolonged exercise events will hamper performance and may lead to serious medical disorders such as heat exhaustion, heat stroke, or hyponatremia. A 1% reduction in body weight due to water loss may evoke undue stress on the cardiovascular system accompanied by increases in heart rate and inadequate heat transfer to the skin and the environment, an increase in plasma osmolality, and a decrease in plasma volume and affect the intracellular and extracellular electrolyte balance [297].

Water loss occurs through respiration, sweat, feces, and urine; however, during prolonged endurance most water is lost in sweat, especially during high environmental temperatures. About 580 kcals are lost for every liter of sweat that is evaporated [298]. Loss of body fluid during endurance exercise can be determined by changes in body weight; each kg of body weight loss accounts for about 1 liter of fluid loss. Sports drinks with adequate concentrations of electrolytes and carbohydrates promotes maintenance of homeostasis, prevents injuries, and maintains optimal performance [299].

*Mechanisms*. Regulation of fluid balance is a remarkably complex process. Water is lost from the body through the skin, feces, lungs, and kidneys. Water retention by the kidneys is directly controlled by vasopressin produced in the hypothalamus. Production of vasopressin is affected by hypothalamic receptors sensitive to plasma osmolarity and stretch receptors in the atria of the heart, carotid arteries, and aorta.

The kidneys actively reabsorb sodium to regulate extracellular fluid osmolarity and this is largely controlled by aldosterone produced by the adrenal cortex. As serum osmolarity decreases, the adrenal cortex release of aldosterone is triggered resulting in more sodium reabsorbed and an increase in osmolarity. The kidneys also regulate aldosterone production through the rennin-angiotensin mechanism. Receptors in the juxtaglomerular complex of the kidney tubules respond to low volume (pressure) by releasing rennin, which leads to a hormonal cascade effect resulting in production of angiotensin II, a potent vasoconstrictor, which stimulates the release of aldosterone.

Prolonged E exercise significantly taxes the body's ability to regulate hydration status, body temperature, and electrolytes, thus maintaining hydration during exercise is critical to optimal performance. It is recommended that athletes ingest ~500 mL of fluid 1-2 hours prior to performance and continue to consume cool drinks in the amount of 4–6 ounces every 20 minutes during exercise to replace sweat losses [297, 300, 301]. The rate of water ingestion should not exceed the rate of water loss, as it might result in water retention, weight gain, and exercise-associated hyponatremia [301, 302].

*Evidence*. Consumption of sports beverage drinks during exercise is recommended to meet carbohydrate energy needs and to replace sweat, water, and electrolyte losses [297]. The majority of the literature supports fluid, carbohydrate, and electrolyte replacement during prolonged (≥60 minutes) endurance exercise. Replacement of Na^+^ and K^+^ are essential to maintain plasma volume and hydration [303]. Different exercise tasks (metabolic requirements, duration, clothing, and equipment), weather conditions, and other factors such as genetic predisposition, heat acclimatization, and training status influence sweating rate and electrolyte concentrations and determine fluid needs [297].

Carbohydrate and electrolyte content, palatability, color, odor, taste, temperature, and texture of a sports drink can increase fluid consumption before, during, and after exercise [297, 304] and therefore improve performance. Athletes should ingest 4 to 8 ounces of a 6%–8% carbohydrate-electrolyte sports drink every 10 to 20 minutes during exercise and avoid carbohydrate concentrations over 8% as this will delay gastric-emptying and should be avoided. Increasing plasma volume can positively affect performance and sodium in sports drinks may help achieve this by improving glucose and water absorption in the small intestine. Sodium is important in rehydration, especially during exercise in the heat [305].

Galloway and Maughan [306] studied six healthy males who cycled to exhaustion while ingesting either no drink, a 15% carbohydrate-electrolyte drink, or a 2% carbohydrate-electrolyte drink. Consumption of the 2% carbohydrate-electrolyte drink leads to a lower serum osmolality and reduced plasma volume deficits. Potassium is important in rehydration after exercise due to the increased retention of fluid in the intracellular space [305]. Numerous recent studies [305, 307–310] have confirmed that during E events, consumption of glucose-electrolyte solutions improved performance greater than water alone.

Several factors including fluid, fuel substrate, and electrolyte depletion have been implicated in the reduction of endurance performance. Recent investigations have suggested that consumption of lactate and fructose in energy-electrolyte hydration beverages improves performance and delays fatigue compared to glucose-electrolyte beverages via increased substrate oxidation and enhanced buffering capacity [311].

Hyperhydration may be induced by the oral consumption of glycerol which induces an osmotic gradient that favors greater renal water absorption. Studies examining the effect of hyperhydration by glycerol consumption on performance are equivocal. Several studies have shown performance enhancements [298, 312, 313] while others have shown no difference when comparing hyperhydration by glycerol consumption to hyperhydration by water or flavored-water consumption [314–316]. Recently, investigations have examined the effect of glycerol ingestion and fluids of varying tonicities (0.9% versus 0.45% NaCl) during the rehydration period following exercise-induced dehydration (~4% of body weight) and prior to exercise in the heat [307, 317]. Rehydration with either a 0.45% or 0.9% NaCl solution resulted in similar fluid restoration, similar cardiovascular, thermoregulatory, and exercise performance responses and were superior to no fluid ingestion [317]. Glycerol ingestion during the rehydration period was found to significantly prolong subsequent exercise time to exhaustion in the heat but was not associated with specific thermoregulatory or cardiovascular advantages compared to rehydration with water alone [307].

*Practical Use*. The most recent data suggest that multiple-transportable-carbohydrates containing a combination of glucose/maltodextrin + fructose in combination with electrolytes are the most favorable beverages to ingest during endurance exercise to enhance performance. Specifically, a 6–8% carbohydrate mixture of glucose and fructose (GF) plus an electrolyte solution containing NaCl and K will further aid endurance performance [318]. Coconut water is also gaining in popularity due to its high K concentration. Mixing the coconut water with a GF plus NaCL solution may serve as another electrolyte beverage to enhance hydration and performance.

### 6.5. Modified and Resistant Starches

As previously mentioned (see carbohydrate intake), endurance athletes must maintain blood glucose and replenish glycogen stores during and following longer bouts, respectively [92, 273]. Indeed, the type of CHO (glycemic index and gastric-emptying rate) in relation to the timing of exercise (pre- and during exercise) is critical in the maintenance of blood glucose and insulin, sparing hepatic glycogen stores, and manipulating substrate utilization for endurance exercise. The blood glucose and insulin responses vary depending on CHO digestion and gastric-emptying rate and need to be considered prior to competition. In efforts to minimize and control the spike in blood glucose and insulin from CHO intake prior to exercise, research has turned to the use of modified and resistance starches as CHO alternatives.

*Mechanism*. Modified starches have gained popularity because of the benefits to digestion and gastric-emptying rate mostly due to the amylose : amylopectin ratio. In general, the higher the ratio of amylose : amylopectin, the greater the resistance to digestion [319], blunting the initial response of blood glucose and insulin. This spares glycogen stores and enhances fat oxidation. However, it should be noted that despite the amylose : amylopectin ratio the gastric emptying and absorption rates may also be manipulated by modifying the different starches consumed (i.e., hydrothermal modification) [253].

*Evidence*. Stephens et al. [320] measured the effects of a high molecular weight (HMW) rapidly digested modified starch commercially known as Vitargo, a low molecular weight glucose polymer (LMW) (similar to commercial sports drinks), and sugar-free water (SFW) on blood glucose and insulin for two hours after a glycogen depleting exercise (GDE) (60 min at 75% of VO_2max⁡_). Following the two-hour postprandial period each individual performed a 15 min “all-out” bout of cycling. Both the HMW and LMW starch elevated blood glucose and insulin during the two-hour recovery versus the SFW, with the initial response (<60 min) of HMW being significantly greater than the LMW. A greater work output (10%) during the 15 min cycle performance was found when consuming HMW compared to LMW and SFW suggesting that the rise in blood glucose and insulin allowed replenishment of glycogen stores from the HMW between exercise sessions [320].

Jozsi et al. [321] tested blood glucose and insulin response to amylose and amylopectin versus glucose and maltodextrin. Amylose in the form of a resistance starch (see Section 7) acts similar to a dietary fiber allowing increased fat oxidation by blunting glucose and insulin prior to exercise, whereas amylopectin (as waxy maize starch) responds similar to a normal CHO (i.e., glucose). In their study [321] male cyclists (*n* = 8) completed a GDE (60 min at 75% of VO_2max⁡_) where they consumed either resistance starch (100% amylose), waxy maize (100% amylopectin), glucose, or maltodextrin as 65% (~1950 kcals) of a 3,000 kcal diet for twenty-four hours after GDE. Following the GDE and CHO consumption the individuals performed a thirty-minute cycling time trial. Twenty-four hours after the GDE, immediately before exercise the resistance starch (100% amylose) resulted in significantly lower muscle glycogen concentrations compared to waxy maize (100% amylopectin), glucose, and maltodextrin. However, no differences were found between the four types of CHO during the thirty-minute cycling time trial [321]. Therefore, a high amylopectin starch (100%) [321] and a HMW rapidly digesting starch both [320] increase blood glucose and insulin following a glycogen depleting exercise, while high amylose resistant starch (100%) results in a lower blood glucose and insulin response, thereby inhibiting glycogen resynthesis [321].

However, modification of these starches (i.e., hydrothermal modification) may decrease the digestion time altering the response of blood glucose and insulin regardless of the amylose : amylopectin ratio [253]. Roberts et al. [322] measured a hydrothermally modified and slow digesting starch (HMS) consisting primarily of amylopectin (95%), commercially known as UCAN versus maltodextrin (1 g·kg^−1^·BW) during both steady state (150 min submaximal cycling bout at 70% VO_2 peak_) and exhaustive (100% VO_2 peak_) exercise, as well as during 75 minutes of recovery. There was no significant difference in performance between either HMS or maltodextrin. However, both the initial and recovery periods of glucose and insulin were blunted with HMS compared to maltodextrin, allowing an increase in fat oxidation [322]. Thus, despite the HMS consisting of amylopectin (95%) known to increase postprandial glucose and insulin [321], modification of the HMS resulted in a lower postprandial glucose and insulin, similar to that of a high amylose starch [321]. This finding suggests that chemical modification of amylopectin as HMS may augment fat oxidation and spare muscle glycogen.

*Practical Use*. Although performance benefits from modified and resistant starches appear to be minimal, the glucose and insulin responses during exercise and recovery are optimal for both fat oxidation and glycogen resynthesis. Therefore, in efforts to minimize glucose and insulin secretions and promote a greater reliance on fat oxidation it may be recommended to consume (1 g·kg^−1^·BW) either a modified-HMW (UCAN) or 100% amylose starch prior to and during exercise. Athletes who will be competing in multiple events or are dependent on the replenishment of glycogen stores between exercises are recommended a high molecular weight rapidly digesting starch, such as Vitargo. Other whole food sources of resistant starches include cooked and cooled potatoes, whole grains (rice, pasta, etc.), and legumes and, thus, should be consumed prior to and between extended bouts of exercise.

## 7. PEDs for Energy Metabolism and Body Composition

Optimal body composition plays a critical factor in athletic performance and it varies among different types of athletes and sports. It is well known that energy metabolism and body composition are directly related to each other and nutritional factors are the primary determinants of each (Table 4).

### 7.1. Caffeine

The practical use of caffeine, as well as mechanisms through which it may enhance performance and energy metabolism, was previously discussed in relation to I and E athletic performance. In regard to energy metabolism, a meta-analysis including 6 trials found caffeine consumption to increase daily energy expenditure by approximately 100 kcal [323]. However, fat oxidation was found to only increase when caffeine was combined with catechins [323], indicating that tea may be a favorable source of caffeine for the purpose of weight management. In this meta-analysis, caffeine intake ranged from 150 to 1604 mg·d^−1^ [323]. Consistent with the above results, our laboratory found a 5 mg·kg^−1^·FFM dose of caffeine to increase energy expenditure in men [324] and women [325]. Although fat oxidation was not measured in the women, it did not change in the men [324]. In addition, the increase in energy expenditure was reduced for older versus younger women [325], but this was not the case with the men [319, 324] suggesting a gender difference in the influence of age on the metabolic response to caffeine.

In contrast to previous results, two recent trials found 5 mg·kg^−1^·d^−1^·BW of caffeine, consumed for 4 days, to have no influence on resting, active, or total energy expenditure in young men [326, 327]. However, in both trials, the caffeine was divided into two doses, one of which was consumed with breakfast and the other with lunch. Furthermore, although participation was restricted to individuals who habitually consumed less than 100 mg·d^−1^ of caffeine, actual caffeine consumption prior to the trials was not reported. As such, the previously described evidence, indicating that caffeine does increase energy expenditure, appears to be more reliable.

### 7.2. Capsinoids

Capsaicin, the known pungent flavor of hot red chili peppers, has become a popularly marketed natural spice for enhancing thermogenesis (i.e., catecholamines, fat oxidation) and improving satiety [328].

*Mechanism*. Capsaicin will bind to the TRVP1 passively absorbing through the stomach and upper portion of the small intestine. After being released into circulation, capsaicin will be transported to the adrenal gland to release catecholamines, thereby increasing SNSa and energy expenditure [328, 329].

*Evidence*. Research has shown capsaicin to increase SNSa [330–334], energy expenditure [329, 335–337], and substrate oxidation [329, 330, 335, 336, 338], although these findings are not universal [339, 340]. The effectiveness of capsaicin to increase thermogenesis and satiety may differ due to varying dosages and with individuals who frequently consume capsaicin compared to nonusers. More recently, Ludy and Mattes [329] tested the effects of capsaicin (1 g red pepper) on energy expenditure, fat oxidation, and satiety in individuals who regularly consume red peppers versus nonusers in both oral and capsule forms. The postprandial energy expenditure increased in both groups following both oral and capsule form. Interestingly, fat oxidation increased only with the oral form with satiety and energy intake decreasing in only the nonusers. Because capsaicin binds to the TRPV1 in the oral cavity activating heat and pain sensitive sensory neurons it is suggested that when consumed orally rather than in capsule form capsaicin's influence on substrate oxidation may be greatest [329].

Because the pungent sensory burn and pain elicited from capsaicin may cause difficulty in palatability, the capsaicin-like compound capsiate, in the form of nonpungent red pepper “CH-19 Sweet,” is an alternative for those unaccustomed or opposed to eating spices. Despite the difference of activation sites, both capsaicin and capsiate bind with high affinity to TRPV1 located in the gut, increasing SNSa [338, 341] without the elevated systolic blood pressure and heart rate response reported with capsaicin [334]. As documented with capsaicin, similar supporting research has found capsiate to increase SNSa [334, 342], energy expenditure [342–344] and substrate oxidation [338, 342–344] in higher dosages. Josse et al. [342] found increases in SNSa, energy expenditure, and fat oxidation with 10 mg. Likewise, Galgani and Ravussin [345] found a 54 kcal·d^−1^ increase in RMR when individuals consumed 3 and 9 mg compared to placebo, and Lee et al. [344] reported increases in postprandial energy expenditure and fat oxidation with 3 and 9 mg. Thus, when consumed in higher concentrations (3–10 mg) in capsule form, capsiate appears to elicit the greatest thermogenic response.

*Practical Use*. Individuals not accustomed or willing to consume spices, capsaicin, and capsiate may serve as effective PED supplements to augment and optimize postprandial thermogenesis and substrate oxidation. Common sources of capsaicin in market stores are chili powder, chili peppers, cayenne pepper, jalapenos, and habaneros. A lower dosage of 2 mg to a higher dosage of 10 mg is recommended for individuals with varying thresholds and tolerance, with capsiate as an alternative to those unwilling or unable to consume the pungent capsaicin. However, those unaccustomed to eating capsaicin should consider timing because consumption of the spice prior to exercise has been reported to cause stomach discomfort, nausea, intestinal cramping, flatulence, and burning bowel movements in male athletes [346].

### 7.3. Carnitine

Carnitine is naturally synthesized in the body from the essential amino acids lysine and methionine [347]. Based on its role in fatty acid transport, carnitine has the potential to support weight management by facilitating fat oxidation.

*Mechanism*. Carnitine makes up the substrate to CPT1, a rate-limiting step in fatty acid oxidation within skeletal muscle [348]. It is logical to assume that consuming exogenous carnitine via diet or supplementation would be beneficial by inhibiting carbohydrate utilization and augmenting fatty acid oxidation through enhanced translocation of long-chain acyl groups across the inner mitochondrial membrane [348, 349].

*Evidence*. Recently, Stephens et al. [348] tested the effects of 12 weeks of L-carnitine (CAR) in combination with a CHO consumed twice daily on muscle expression of genes associated with metabolism, body composition (DEXA), and energy expenditure during low intensity exercise (50% of VO_2max⁡_ for 30 min) in twelve males. Participants ingested either the CAR + CHO, *n* = 6 (1.36 g + 80 g l-CAR + CHO), or CHO, *n* = 6 (80 g) first thing in the morning and again 4 hours later. Those who consumed CAR + CHO had a 20% and 200% increase in total carnitine and long-chain acyl-CoA, respectively (*P* < 0.05), and elevated expression of genes involved in fatty acid metabolism. There were no changes in body composition in the CAR + CHO group; however over 12 weeks there was a 1.9 and 1.8 kg increase in body mass and whole-body fat mass, respectively, in the CHO group. Furthermore, there were no changes in whole-body energy expenditure in the CHO group, but there was a significant increase of 6% in the CAR + CHO group during the 30 min low-intensity exercise. These findings from Stephens et al. [348] suggest that the consumption of CAR + CHO for twelve weeks prevented increased body fat mass and in turn was associated with a greater energy expenditure and fat oxidation during low-intensity exercise, as supported in previous research [350].

*Practical Use*. Carnitine is found in abundance throughout the skeletal muscle cells of the body [351] and may be ingested through diet containing red meat, fish, poultry, and dairy products (for amount of carnitine per each nutrient, see Rebouche [352]) or can be biosynthesized within the liver and kidneys primarily from the essential amino acids lysine and methionine [351, 352]. Those who consume a vegetarian diet are estimated to receive ~90% of their total available carnitine from endogenous synthesis due to the lack of available carnitine in plant foods, while omnivores receive one-eighth to one-half of total carnitine through diet [352]. Therefore athletes consuming a predominately plant based diet may consider commercially produced carnitine supplements which have been shown to be safe in humans [353].

### 7.4. Dietary Fiber and Resistant Starches

Resistance starches (RS) are touted as weight loss wonder foods because they have digestive properties and satiating effects similar to those of dietary fibers [354]. In addition, RS may further facilitate weight management by increasing fat oxidation and total energy expenditure and improving glycemic regulation [354].

*Mechanism*. In comparison to normal dietary starches (DS), RS lowers the glycemic response by passing digestion in the small intestine and moving directly into the large intestine, where it is fermented into short-chain fatty acids [354, 355]. Of the five types of RS1, RS2, RS3, and RS4 are most commonly measured in humans for their effects on postprandial glycemia/insulinemia responses and gut satiety peptides that influence weight loss or weight maintenance (i.e., energy expenditure). RS1 is physically inaccessible to digestive enzymes from the presence of seed coats (e.g., whole grains), RS2 is a high amylose maize starch comprised primarily of *α*-1,4 glycosidic links, RS3 is retrograded starch (e.g., pasta or rice that has been cooked then cooled), and RS4 is chemically modified to be resistant to digestion [354, 356].

*Evidence*. Following consumption of RS2, the postprandial glycemia/insulinemia responses in healthy men and women have been shown to significantly decrease compared to DS [357, 358]. Interestingly though, when RS2 was adjusted as 0%, 2.7%, 5.4%, and 10.7% (percentage of total carbohydrate) at 30% of an individuals' daily energy needs there were no differences in postprandial glycemia/insulinemia, suggesting dosages up to 10% have little impact on glycemia and that a combination of additional ingredients might affect RS2 function [359]. Among the other types of RS, RS3 has been shown to decrease postprandial glycemia [360], and RS4 has resulted in decreases in both postprandial glycemia and insulinemia [361–363].

In addition to improved postprandial glycemic/insulinemic responses, RS2 and 3 have shown to positively alter gut satiating peptides (glucose-dependent insulinotropic peptide (GIP)) [358], suppress energy intake [357], improve satiation/appetite [358, 364], and significantly increase fat oxidation [359], although these findings are not universal [357, 359, 365, 366]. Yet, despite the conflicting results of RS2 and RS3, RS4 has consistently reported favorable postprandial glycemia/insulinemia responses [355, 363, 367] and beneficial increases in energy expenditure in healthy individuals [363].

To determine differences in postprandial glycemia/insulinemia responses between RS (RS2 and RS4 cross-linked, XL) and a normal carbohydrate (dextrose), Haub et al. [355] tested eleven healthy males (*n* = 4) and females (*n* = 7) for two hours after consuming 30 g of RS4_XL_, RS2, or dextrose combined with water. Postprandial glucose and insulin responses were significantly lower in the RS4_XL_ and RS2 compared to dextrose, with RS4_XL_ being significantly lower than that of the RS2 [355]. Because RS is more commonly consumed in combination with foods rather than water alone, Al-Tamimi et al. [367] examined the effects RS4_XL_ in combination with additional ingredients in the form of a nutrition bar and found that RS4_XL_ compared to wheat starch resulted in significantly lower 2 hr postprandial glycemia/insulinemia response.

Shimotoyodome et al. [363] tested RS4 (hydroxypropyl-distarch, HDP) versus waxy maize starch (WMS) using a pancake meal on the 3 hr postprandial glycemic/insulinemic, GIP, and energy expenditure response in healthy lean males. HDP resulted in significantly lower postprandial glycemia/insulinemia and GIP, as well as increased fat oxidation and energy expenditure compared to WMS. Research supports suppressed postprandial glycemia/insulinemia by both RS2 and RS4; interestingly, only RS4 has elicited significant increases in energy expenditure [363].

*Practical Use*. Current research suggests the effects of RS4 (RS4_XL_ and HDP) on postprandial glycemia/insulinemia, gut satiety peptides GIP, and augmented energy expenditure and fat oxidation [355, 363, 367] are greater than those of RS2. A suggested dosage of 20–40 g of RS4 consumed at breakfast or a late evening snack may facilitate greater appetite suppression, postprandial glycemia and insulinemia, and increase energy expenditure.

### 7.5. Medium-Chain Triglycerides

Medium-chain triglycerides (MCTs) consist of fatty acids ranging in length from 6 to 12 carbons [368]. Although MCTs appear to have little influence on performance, benefits related to energy balance and weight loss are better supported [369].

*Mechanisms*. The benefits of MCT consumption may be explained by the unique ways their constituent fatty acids are absorbed and metabolized. Prior to reaching systemic circulation, long-chain fatty acids are reincorporated into triglycerides, assembled into chylomicrons, and released into lymphatic circulation [370]. In contrast, medium-chain fatty acids enter portal circulation directly through the enterocyte [370]. As such, medium-chain fatty acids enter circulation more rapidly and are primarily absorbed by the liver [368]. Once absorbed, medium-chain fatty acids pass through the inner mitochondrial membrane independently of the CPT transport system and can therefore be rapidly oxidized [368]. This, along with the poor binding potential between medium-chain fatty acids and fatty-acid-binding protein, limits the lipogenic potential of MCTs [368]. As such, MCTs are more likely to be utilized for energy and less likely to be stored as body fat.

MCTs also have the potential to increase energy expenditure [371]. Urinary noradrenaline excretion has been found to increase in conjunction with increased energy expenditure following MCT consumption [372]. Furthermore, in rats fed MCTs, an increase in energy expenditure was prevented with administration of propranolol [373]. Therefore, increased SNSa may be the underlying mechanism.

*Evidence*. In a recent systematic review [371], 6 of 8 trials found MCT consumption to improve body composition, and 4 of 6 trials identified an increase in energy expenditure [371]. However, an increase in satiety was only observed in 1 of 7 trials [371]. Some trials have shown MCT consumption to increase average daily energy expenditure by more than 100 kcal in overweight [374] and normal-weight [372] men, which could amount to more than 30 lbs of weight loss over a year [375]. However, such an increase is not consistently supported [371] and may be less notable in women [376]. Furthermore, the increase in energy expenditure induced by MCT consumption has been shown to diminish over time [369]. Despite this, superior weight loss has been observed in trials lasting as long as 16 weeks [369]. However, because the increases in energy expenditure and fat oxidation associated with MCT consumption have been inversely correlated with initial body weight, MCT consumption may be more effective for preventing weight gain than promoting weight loss [377].

In a recent trial including 7 normal-weight subjects, a breakfast meal containing 20 g of MCTs was found to increase diet-induced thermogenesis and fat oxidation compared to the same meal with a calorically matched content of sunflower oil [378]. Similarly, in another recent trial, the inclusion of MCTs in a meal replacement shake led to greater diet-induced thermogenesis compared to shakes with lesser amounts of MCTs or no MCTs at all [379]. As such, replacing a portion of dietary fat with MCTs may be an effective strategy for weight maintenance.

*Practical Use*. Increases in energy expenditure have been observed with MCT intakes ranging from 8 to 35 g·d^−1^ [371]. While MCT oil is readily available, coconut oil and palm kernel oil are two alternatives that more closely resemble whole foods. They contain approximately 63 and 58% MCT, respectively [380]. These oils can easily be incorporated into the diet through cooking or by melting them for use as a sauce or salad dressing. The meat of a coconut, although less practical, is truly a whole food and contains approximately 19% MCT [94].

## 8. Summary

Advances in athletic performance training and nutrition have prompted a reevaluation of our current practices in order for both (training and nutrition) to work synergistically with each other instead of in isolation to one another. The current review, albeit novel, bridges the gap between athletic performance training and sports nutrition by linking the scientifically validated multicomponent training model (timed-protein feedings; resistance training; interval sprint training; stretching/recovery training; and endurance training; PRISE) employed by most, if not all, athletes with specific performance enhancing diets (PEDs) to foster optimal athletic performance. The goal of this innovative review is to provide a new paradigm of sports nutrition that allows performance training (PRISE) and sports nutrition (PEDs) to complement each other instead of working apart from one another.

## Figures and Tables

**Table 1:** PRISE protocol.

	Exercise	Type	Work	RPE	Monday	Tuesday	Wednesday	Thursday	Friday
PRISE	Protein-pacing (P)	P, A	—	—	20 grams × 5 servings	20 grams × 5 servings	20 grams × 5 servings	20 grams × 5 servings	20 grams × 5 servings
	Resistance (R)	WB	2 sets/exercise 10–15 reps	7–9	WB	—	REST	—	—
	Intervals (I)	C	5–7 sets 30 s/4 min rest	10/3	—	X		—	—
	Stretching (S)	S	≤60 min	7–9	—	—		WB	—
	Endurance (E)	C	≥60 min	6	—	—		—	X

Note: P: plant-based; A: animal-based; RPE: rating of perceived effort; RT: resistance training; Sprint: sprint interval training; C: choice of exercise modality; WB: whole body exercise; S: stretching exercise; X: exercise day. Exercise modalities available for C include walking, jogging, running, cycling, swimming, elliptical, rowing, rollerblading, and cross-country skiing.

**Table 2:** Resistance exercise (R).

Circle the exercises performed from each category			Reps/time	Resistance
Dynamic warm-up	Perform prior to each workout (5–10 minutes):			
	(1) Pendulum swings (side-to-side)	(7) Over-under the fence		
	(2) Pendulum swings (front-to-back)	(8) Hip opening/closing		
	(3) High knee (chest)	(9) High knees		
	(4) High knee (external rotation)	(10) Butt kicks		
	(5) Side shuffle	(11) Lunge with twist		
	(6) Carioca	(12) Arm windmills		

Footwork and agility	Perform using agility ladder (10 minutes):			
	(1) Forward, double-step	(1) Side shuffle		
	(2) Sideways double-step	(2) Figure 8's		
	(3) Side-step, double in/out	(3) Kangaroo hops 2/1 foot		
	(4) Side shuffle, two-in/out	(4) Kangaroo hops, sideways		
	(5) Two leg hops	(5) T-drill		
	(6) One leg hops	(6) Jump rope		
	(7) Two leg hops, in/out			
	(8) One leg hops, in/out			
	(9) One leg hops, sideways			

Resistance and power exercises	Perform each below (10 minutes):	Perform each below (10 minutes):		
	(1) Side-steps toes in/out, ankles/knees	(1) Back rows/flys		
	-Side-steps with bands and med ball	(2) Pull-ups		
	(2) Forward/backward walk with bands	(3) Chest press/fly		
	(3) Squats	(4) Pushups (choose one):		
	(4) Lunges with tubing (with med ball)	(i) Side walking		
	(5) Lateral lunges (with med ball)	(ii) Knees/toes w/physioball		
	Choose 2 below:	(iii) Down dog		
	(6) Front step-ups	(iv) Side to side (ball)		
	(7) Squat thrusts, med ball throws	(v) Heart-to-heart		
	(8) Jump squats	(vi) Hi/low		
	(9) Mountain climbers	(5) Front/lateral raises		
	(10) Squat-plank-jump squats	(6) Biceps curls		
	(11) Lateral step-ups	(7) Shoulder press		
		(8) Hyperextensions		

Core Exercises	Perform 4 below (10 minutes):	Perform 4 below (5 minutes):		
	(1) Plank knees elbows/hands	(1) Knees to chest		
	(2) Plank toes elbows/hands	(2) Hyperextension on ball		
	(3) Plank one leg elbows	(3) Reverse planks		
	(4) Plank one leg hands on ball	(4) Ab hollow		
	(5) Side planks foot-elbow/twist	(5) Walking sit-ups		
	(6) Side planks hand stars	(6) Crunch bent knee		
	(7) Airplanes	(7) Tug-of-war		
	(8) Supermans/womans	(8) Side touch/scissors/toe		
	(9) Crunches on ball			
	(10) Plank with ball on knees/toes			

Resistance exercises utilize medicine balls, physioballs, rubber tubes and bands which are incorporated into a dynamic warm-up, footwork and agility drills, resistance and power movements, and core exercises, bodyweight exercises (e.g., lunges, squats, and jumping rope). A 5 minute cool down follows the R routine with gentle stretching. Total R exercise time is 60 minutes.

**Table 3:** Stretching exercise (S).

Circle the exercises performed from each category		Breaths/time
Sun salutations	(1) Mountain pose (*Tadasana*)	
	(2) Standing forward bend (*Uttanasana*)	
	(3) Plank pose (*Phalakasana*)	
	(4) Four-limbed staff pose (*Chaturanga Dandasana*)	
	(5) Cobra pose (*Bhujangasana*)	
	(6) Upward facing dog pose (*Urdhva Mukha Svanasana*)	
	(7) Downward facing dog pose (*Adho Mukha Svanasana*)	
	(8) Child's pose/rest pose (*Balasana*)	

Standing poses	(1) Neck stretching	
	(2) Side bending	
	(3) Lunge pose (*Anjaneyasana*)	
	(4) Warrior I pose (*Virabhadrasana I*)	
	(5) Warrior II pose (*Virabhadrasana II*)	
	(6) Triangle pose (*Utthita Trikonasana*)	
	(7) Extended side angle pose (*Utthita Parsvakonasana*)	
	(8) Goddess pose (*Utkata Konasana*)	
	(9) Chair pose (*Utkatasana*)	
	(10) Revolved chair pose (*Parivrtta Utkatasana*)	
	(11) Squat pose (*Malasana*)	
	(12) Standing wide-legged forward bend pose (*Prasarita Padottanasana*)	

Balance in motion poses	(1) Tree pose (*Vrksasana*)	
	(2) Warrior III (*Virabhadrasana III*)	
	(3) Lord of the dance pose (*Natarajasana*)	
	(4) Standing one-legged balance	
	(5) Eagle pose (Garudasana)	
	(6) Boat pose (*Navasana*)	
	(7) Bicycle pose	
	(8) Bow pose (*Dhanurasana*)	
	(9) Candlestick pose	
	(10) Camel pose (*Ustrasana*)	
	(11) Pigeon pose (*Eka Pada Rajakapotasana*)	

Floor poses	(1) Seated cross-legged pose (*Sukhasana*)	
	(2) Staff pose (*Dandasana*)	
	(3) Seated forward bend (*Paschimottanasana*)	
	(4) Head to knee pose (*Janu Sirsasana*)	
	(5) Wide seated forward bend pose (*Upavistha Konasana*)	
	(6) Table top pose and cat/cow	
	(7) Bridge pose (*Setu Bandhasana*)	
	(9) Butterfly pose (*Baddha Konasana*)	
	(10) Happy baby pose (*Ananda Balasana*)	
	(11) Half twist pose (*Ardha Matsyendrasana*)	
	(12) Head to knee pose (*Janu Sirsasana*)	
	(13) Front split pose (*Hanumanasana*)	
	(14) Frog pose (*Mandukasana*)	
	(15) Spinal twist pose (*Supta Matsyendrasana*)	
	(16) Corpse pose (*Savasana*)	

S is based primarily on traditional yoga “asanas,” or poses, with modern elements of Pilates for a total body stretching, flexibility, and strengthening workout. All (S) routines include basic sun salutations, standing poses, balance in motion, a floor core strengthening portion, and a final resting relaxation phase. As participants progress they are instructed to increase the intensity in which they perform the poses so the level of intensity ranges from 7 to 9 on the intensity scale.

**Table 4:** Summary of research supporting the PRISE Protocol of performance enhancing diets for athletic performance.

Author group	Nutrient	Number of participants	Duration (days)	Design	Dose	Performance improvements reported
Resistance						
Antonio and Ciccone, 2013 [55]	Creatine	19	28	Randomized	5 g·d^−1^	(i) Increased lean body mass (ii) Increased 1RM bench press (iii) Supplementation after workout was more effective than before workout
Gouttebarge et al. 2012 [54]	Creatine	16	5	Double-blind, randomized, placebo-controlled	20 g·d^−1^	(i) 2.2% increase in body mass (ii) 2.7% increase in vertical jump peak power
Souza-Junior et al. 2011 [56]	Creatine	22	56	Randomized	20 g·d^−1^ for 7 days 5 g·d^−1^ thereafter (included maltodextrin)	(i) Increased cross sectional area of thigh and arm muscle (ii) Increased 1RM squat and bench press (iii) Comparable results despite reduced training volume due to reduced rest intervals during resistance training
Ispoglou et al. 2011 [87]	Leucine	26	84	Double-blind, placebo-controlled	4 g·d^−1^	(i) Increased 5RM for 5 of 8 resistance exercises

Intervals						
de Salles Painelli et al. 2014 [112]	Beta-alanine	40 20 = BA 19 = PL	4 wks	Double-blind	6.4 g·d^−1^	(i) Increased total work done (ii) Increased mean power output
Ducker et al. 2013 [104]	Beta-alanine	18	28	Randomized, placebo-controlled	80 mg·kg^−1^ BM·d^−1^	(i) Improved 800 m track performance
Van Thienen et al. 2009 [105]	Beta-alanine	17	8 wks	Double-blind	2 g·d^−1^ (days 1–14), then 3 g·d^−1^ (days 15–27), then 4 g·d^−1^ (days 28–56)	(i) Increased sprint performance following a 110 min cycling race
Abian-Vicen et al. 2014 [129]	Caffeine	16	—	Randomized, double-blind, placebo-controlled, crossover	3 mg·kg^−1^ (as part of energy drink)	(i) Increased single and repeated jump height
Del Coso et al. 2014 [127]	Caffeine	15	—	Randomized, double-blind, placebo-controlled, crossover	3 mg·kg^−1^ (as part of energy drink)	(i) Increased single and repeated jump height (ii) Increased ball velocity for volleyball spike (iii) Reduced time to complete agility test
Del Coso et al. 2013 [131]	Caffeine	16	—	Randomized, double-blind, placebo-controlled, crossover	3 mg·kg^−1^ (as part of energy drink)	(i) Increased power output during repeated jumps (ii) Increased running speed during rugby practice games
Del Coso et al. 2013 [132]	Caffeine	26	—	Randomized, double-blind, placebo-controlled, crossover	3 mg·kg^−1^ (as part of energy drink)	(i) Increased number of sprints and distance covered (total and at running speed above 20 km·h^−1^) during a simulated rugby match
Duncan et al. 2014 [134]	Caffeine	10	—	Randomized, double-blind, placebo-controlled, crossover	6 mg·kg^−1^	(i) Increased torque production during isokinetic knee extension at 30, 150, and 300°·s^−1^.
Lane et al. 2013 [133]	Caffeine	12	—		3 mg·kg^−1^	(i) 2.8% increase in mean power output during HIIT with normal glycogen levels (ii) 3.5% increase in mean power output during HIIT with low glycogen levels
Lara et al. 2014 [130]	Caffeine	18	—	Randomized, double-blind, placebo-controlled, crossover	3 mg·kg^−1^ (as part of energy drink)	(i) Increased jump height (ii) Increased sprint speed (iii) Increased number of sprints and distance covered (total and at running speed above 18 km·h^−1^) during a simulated soccer match
Silva-Cavalcante et al. 2013 [135]	Caffeine	7	—	Randomized, double-blind, placebo-controlled, crossover	5 mg·kg^−1^	(i) 4.1% reduction in time to complete 4 km cycling time trial with low glycogen levels (ii) 10.8% increase in mean power output during 4 km cycling time trial with low glycogen levels
Camic et al. 2014 [140]	Creatine (polyethylene glycosylated)	77	28	Randomized, double-blind, placebo-controlled	1.25 g·d^−1^ 2.5 g·d^−1^	(i) Increase in vertical jump height (ii) Increase in bench press endurance (iii) Reduction in times for shuttle-run and 3-cond drill (iv) Increase in body mass
Oliver et al. 2013 [142]	Creatine	13	6	No control group	20 g·d^−1^ (included glucose)	(i) Increased power at lactate threshold (*P* = 0.11), time to fatigue (*P* = 0.056), and maximal power output (*P* = 0.082) during incremental cycling to exhaustion
Zuniga et al. 2012 [141]	Creatine	22	7	Randomized, double-blind, placebo-controlled	20 g·d^−1^	(i) Increased mean power during two Wingate tests separated by 7 minutes
Ducker et al. 2013 [155]	Sodium bicarbonate	24	—	Randomized, blinded, placebo-controlled	0.3 g·kg^−1^	(i) Reduced total, mean, and best times during repeated maximal running sprints
Mero et al. 2013 [156]	Sodium bicarbonate	13	—	Randomized, double-blind, placebo-controlled, crossover	0.3 g·kg^−1^	(i) Reduced time to complete second of 2 maximal 100 m freestyle swims separated by 12 minutes
Mueller et al. 2013 [154]	Sodium bicarbonate	8	5	Randomized, double-blind, placebo-controlled, crossover	0.3 g·kg^−1^	(i) 23.5% increase in time to exhaustion during cycling at critical power (ii) Improved performance maintained throughout 5 consecutive days of supplementation and testing

Stretching						
Black et al. 2010 [177]	Ginger	25	—	Double-blind, crossover study	2 g of raw 2 g of heat-treated	(i) Decreased perception of pain following eccentric exercise
Chuengsamarn et al. 2014 [178]	Curcumin	213 107 = curcumin 106 = placebo	6 months	Randomized, double-blind, placebo-controlled	250 mg per capsule 6 capsules per day	(i) Decreased pulse wave velocity (ii) Increased adiponectin (iii) Decreased leptin (iv) Decreased HOMA-IR, triglyceride, uric acid, visceral, and total body fat
Takahashi et al. 2014 [191]	Curcumin	10	—	Double-blind, placebo-controlled, counterbalanced crossover	90 mg-single and placebo 180 mg-double	(i) Decreased reactive oxygen metabolites in both groups versus placebo (ii) Increased biological antioxidant potential concentrations in both groups versus placebo
Bloomer et al. 2009 [194]	Omega-3 (EPA : DHA)	14	6 wks	Random order double-blind crossover design study	EPA : DHA 2,224 : 2,208 mg·d^−1^,	(i) Decreased resting levels of inflammatory biomarkers (C-reactive protein and TNF-*α*)
Tartibian et al. 2009 [195]	Omega-3 (EPA : DHA)	27 (*n* = 9, control) (*n* = 9, placebo) (*n* = 9, EPA : DHA)	32	Randomized, double-blinded, repeated measures	324 : 216 mg·d^−1^, 30 days and 48 hrs during recovery	(i) Decreased perceived pain and ROM at 48 hours postexercise
Jouris et al. 2011 [196]	Omega-3 (EPA : DHA)	11	7	Repeated measures intervention	2,000 : 1,000 mg·d^−1^ for 7 days	(i) Decreased perceived muscle soreness, pain, and swelling.
Smith et al. 2011 [199]	Omega-3 (EPA : DHA)	16	8 wks	Randomized controlled study	EPA : DHA 1.86 : 1.50 g·d^−1^	(i) Stimulating protein synthesis through activation of the mTOR-p70s6k signaling pathway in older adults

Endurance						
Bailey et al. 2009 [218]	Beet root juice	8	6	Double-blind, placebo- (PL-) controlled, crossover study	0.5 liters of BRJ (5.5 mmol/day of NO_3_^−^)	(i) Single dose BRJ lowered VO_2_ during submaximal exercise of 60% maximal work rate (ii) BRJ significantly improved 16.1 km TT performance
Vanhatalo et al. 2010 [219]	Beet root juice	8	15	Balanced crossover	0.5 liters BRJ (5.2 mmol/day NO_3_^−^)	(i) VO_2_ max, peak power output, and work rate associated with anaerobic threshold were higher than placebo and baseline after 15 days of BRJ
Lansley et al. 2011 [217]	Beet root juice	9	6	Randomized, double-blind, crossover design	0.5 liters of BRJ (6.2 mmol/day of NO_3_^−^)	(i) Reduced the VO_2_ for constant-work-rate moderate and severe-intensity running by ~7% (ii) Time to exhaustion was increased during severe-intensity running by ~15% and incremental knee-extension exercise by ~5%
Lansley et al. 2011 [216]	Beet root juice	9	—	Randomized, crossover	0.5 liter BRJ (6.2 mmol of NO_3_^−^)	(i) Reduced time to completion and significantly increased power output during the 4 km TT (2.8% and 5%, resp.; *P* < 0.05) (ii) Reduced time to completion and significantly increased power output during the 16 km TT (2.7% and 6%, resp.; *P* < 0.05)
Kenjale et al. 2011 [231]	Beet root juice	8	—	Randomized, open-label, crossover study	0.5 liters of BRJ (18.1 mmol/L NO_3_^−^)	(i) Increased exercise tolerance (walked 18% longer before claudication pain onset and experienced a 17% longer peak walking time) (ii) Decreased fractional O_2_ extraction (48% decrease in Hgb peak-curve amplitude)
Murphy et al. 2012 [226]	Beet root juice	11	—	Double-blind placebo-controlled crossover	200 g Beetroot with ≥500 mg NO_3_^−^	(i) Nonsignificant improvement in running velocity (ii) Running velocity was 5% faster during the last 1.1 miles (1.8 km) of the 5-km run
Hodgson et al. 2013 [137]	Caffeine	8	—	Randomized, single-blind, placebo-controlled, crossover	5 mg·kg^−1^	(i) 4.9% reduction in cycling time until completion of 70% of maximal work output (ii) Comparable results with coffee as the source of caffeine
Pitchford et al. 2014 [234]	Caffeine	9	—	Randomized, double-blind, placebo-controlled, crossover	3 mg·kg^−1^	(i) Reduced cycling time to complete work-based time trial in hot conditions (*P* = 0.06)
Spence et al. 2013 [233]	Caffeine	10	—	Randomized, double-blind, placebo-controlled, crossover	200 mg	(i) Reduction of cycling time during second half of 40 km time trial (ii) Insignificant 1.3% reduction in total cycling time during 40 km time trial
Stadheim et al. 2013 [232]	Caffeine	10	—	Randomized, double-blind, placebo-controlled, crossover	6 mg·kg^−1^	(i) 4% reduction in time to complete 8 km cross-country skiing double-poling time trial (ii) Reduced rating of perceived exertion during 5 minute warm-up intervals at 40, 50, 60, and 70% of aerobic capacity
Stephens et al. 2008 [320]	LMW HMW	8	—		100 g LMS, HMS, or P	(i) Increased performance in LMS and HMS versus placebo (ii) Increased performance in HMS versus LMS
Roberts et al. 2011 [322]	HMS MAT	9	—	Crossover, randomized, double-blind	1 g/kg BM MS 1 g/kg/MD	(i) Decreased glucose and insulin in HMS versus MAT (ii) Increased fat breakdown in HMS versus MAT

Body composition						
Ludy and Mattes 2011 [329]	Capsaicin	25	—	Randomized, crossover	1 g RP after high-FAT diet 1 g RP after high-CHO diet 0 after high-FAT diet 0 after high-CHO diet	(i) Increased EE, core body temperature, and fat oxidation (in oral form) (ii) Decreased energy intake in nonusers, but no Δ in users
Yoneshiro et al. 2012 [337]	Capsaicin	18	—	Single-blind, randomized, placebo-controlled, crossover	9 mg capsinoids (capsules) with 199 mg of rapeseed oil and medium-chain triglycerides 0 (Placebo)	(i) Increased EE through activation of brown adipose tissue in humans
Galgani and Ravussin 2010 [345]	Capsiate	78	4 wks	Parallel-arm double blind, randomized	3 mg·d^−1^ dihydrocapsiate (capsules) 9 mg·d^−1^ dihydrocapsiate (capsules) 0 (Placebo)	(i) Increased RMR when both groups 3 and 9 mg·d^−1^ were combined
Josse et al. 2010 [342]	Capsiate	12	—	Randomized, crossover, double blind	10 mg capsinoids (capsules) 0 (Placebo)	(i) Increased SNSa, energy expenditure, and fat oxidation
Lee et al. 2010 [344]	Capsiate	46	4 wks	Parallel-arm double blind, randomized	3 mg·d^−1^ dihydrocapsiate (capsules) 9 mg·d^−1^ dihydrocapsiate (capsules) 0 (Placebo)	(i) Increased energy expenditure 9 mg·d^−1^ and 3 mg·d^−1^ versus placebo and 9 mg·d^−1^ versus 3 mg·d^−1^
Snitker et al. 2009 [338]	Capsiate	80	12 wks	Parallel-arm double blind, randomized	6 mg·d^−1^ capsinoids (capsules) 0 (Placebo)	(i) Decreased abdominal adiposity (ii) Tended to increase fat oxidation
Inoue et al. 2007 [343]	Capsiate	44	4 wks	Parallel-arm double blind, randomized	3 mg·d^−1^ capsinoids (capsules) 10 mg·d^−1^ capsinoids (capsules) 0 (Placebo)	(i) Increased VO_2_ (10 mg, BMI ≥25 kg/m^2^)
Stephens et al. 2013 [348]	Carnitine	12	12 wks	Randomized, double-blind	1.36 g L-carnitine + 80 g of CHO 80 g of CHO	(i) Increased muscle carnitine by 20% (ii) Prevented an 18% increase in body fat mass found with the CHO group alone (iii) Increased EE and fat oxidation during low-intensity exercise
Haub et al. 2010 [355]	Resistant starch	11	—	Single-blind randomized, crossover	30 g RS4_XL_ 30 g RS2 30 g DEX	(i) Lower plasma glucose for RS4_XL_ and RS2 than DEX, and for RS4_XL_ than RS2
Al-Tamimi et al. 2010 [367]	Resistant starch	13	—	Randomized, crossover	75 g GLU 65 g of puffed wheat bar (PWB) 80 g of RS4_X_	(i) Lower glucose 20–60 min and insulin 30–120 min in RS4_XL_ versus PWB and GLU
Shimotoyodome et al. 2011 [363]	Resistant starch	10	—	Randomized, crossover	38 g RS4-HDP 38 g RS2-WMS	(i) Lower glucose and insulin, and GIP (ii) Increased fat oxidation and EE

HMW: high molecular weight; LMW: low molecular weight; HMS: hydrothermally modified starch; MAT: maltodextrin.
